# A jumbo cyanophage encodes the most comprehensive ribosomal protein set in the known virosphere

**DOI:** 10.1093/ismejo/wrag084

**Published:** 2026-04-10

**Authors:** Isaac Meza-Padilla, Sarit Avrani, Kirsten M Müller, Jozef I Nissimov

**Affiliations:** Department of Biology, University of Waterloo, 200 University Avenue West, Waterloo, ON N2L 3G1, Canada; Department of Evolutionary and Environmental Biology and Institute of Evolution, University of Haifa, Haifa 3498838, Israel; Department of Biology, University of Waterloo, 200 University Avenue West, Waterloo, ON N2L 3G1, Canada; Department of Biology, University of Waterloo, 200 University Avenue West, Waterloo, ON N2L 3G1, Canada

**Keywords:** Ribosome, cyanophage, jumbo phage, prophage, *Vampirovibrio*, *Microcystis*, horizontal gene transfer, paleovirology, virus ecology, virus evolution

## Abstract

It has been proposed that a defining distinction between viruses and cells lies in the absence or presence of ribosomal genes, respectively. Recent studies revealing that viruses occasionally encode ribosomal proteins (RPs) have challenged this view. However, so far, only viral genomes with up to three RPs have been discovered. Here, we perform a functional genome analysis of the *Microcystis* jumbo phage PhiMa05 and show that it encodes six RPs, an RP acetyltransferase, and a ribosome biogenesis protein. To our knowledge, this makes PhiMa05 the first cyanophage reported to encode RPs, as well as the virus with the most comprehensive RP-coding set of the known virosphere. Evolutionary analyses suggest that these viral RP-coding genes may have been horizontally transferred from a temperate ancestor of PhiMa05 to certain members of the *Vampirovibrionia*, a non-photosynthetic basal lineage of *Cyanobacteriota*, via the integration of the viral genome. We find that four RPs, the RP acetyltransferase, and the ribosome biogenesis protein of the PhiMa05-like prophages are the only copies of those proteins that the near-complete genomes of some *Vampirovibrio* hosts possess. We hypothesize that such cellular organisms may depend on the PhiMa05-like prophage for protein synthesis, and hence life itself. Collectively, our results provide evidence for the existence of viruses with particularly enriched sets of RP-coding genes and indicate that, in some cases, such viral genes have been transferred to cells, potentially becoming essential for the survival of the host.

## Graphical Abstract

**Graphical Abstract:**
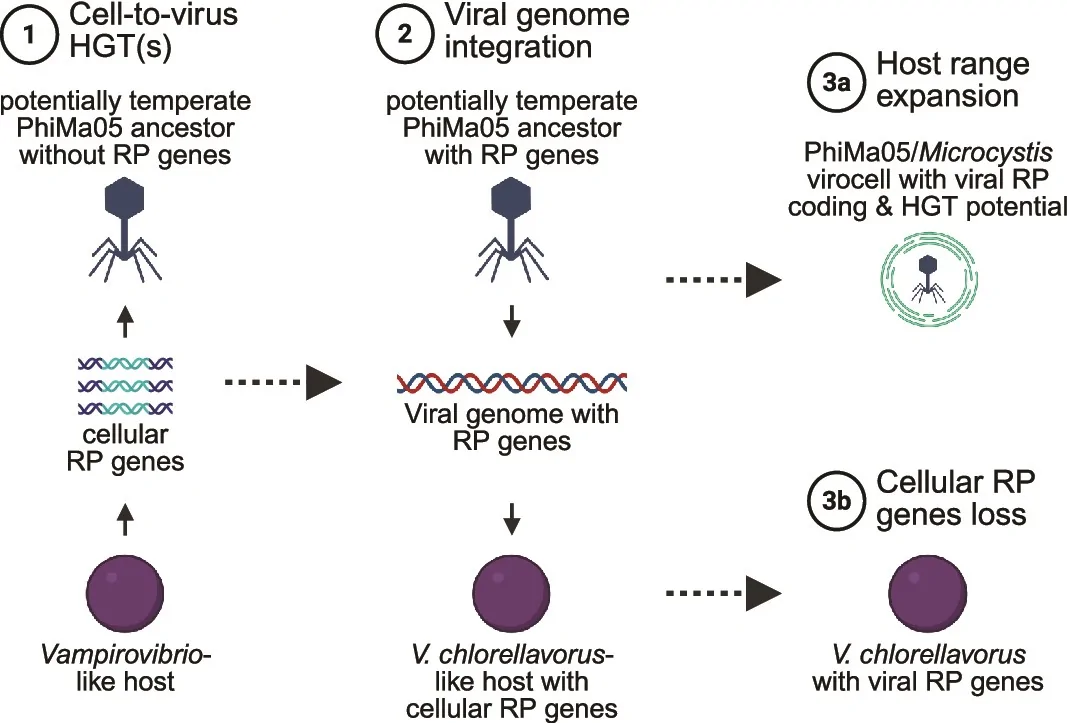

## Introduction

A defining distinction between viruses and cells has been proposed to be the absence or presence of ribosomal genes, respectively [1]. This view was recently challenged by the discovery that viral genomes (including jumbo phages) occasionally encode ribosomal proteins (RPs) [2–9]. However, when they do, viruses usually harbor a single RP-coding gene [2–9]. Even giant Tupanviruses, possessing the most complete translational apparatus of the known virosphere, lack RPs altogether [10]. Thus far, to our knowledge, the viral RP-coding record has been held by an uncultivated Megagenomovirus encoding three RPs [9]. Collectively, these intriguing findings prompt the following question: Are there viruses in which selection has particularly favored the acquisition of RP-coding genes?

PhiMa05 is a lytic jumbo phage that contains a ~274 kb linear double-stranded DNA genome with 256 open reading frames (ORFs) and was isolated from an aeration basin of the wastewater treatment plant of a hospital in Thailand [11]. This environmentally and societally relevant virus infects seven toxic, harmful algal bloom-forming *Microcystis* strains isolated from the eutrophic, fresh-brackish-saline water Songkhla Lake [11, 12]. Whole-genome proteomic [13], major capsid protein [11], and portal protein (Fig. S1) phylogenies place PhiMa05 among freshwater cyanophages. This is consistent with the freshwater nature of its host, *Microcystis*. Our phylogenetic reconstruction (Fig. S1) strongly supports a close relationship between PhiMa05 and the *Saffermanviridae* family, a freshwater clade of temperate cyanophages comprised of free viruses and prophages [14].

In the study by Naknaen *et al.* [11] examining the PhiMa05 genome, 54 ORFs were functionally annotated, however none were identified to be RPs (Table S1). Here, we conduct a functional genome analysis of PhiMa05 and find that it encodes the largest number of RPs discovered in a viral genome to date. We then explore the evolutionary trajectory of the RPs encoded by PhiMa05, elaborating on the ultimate causes and consequences of this set of viral proteins.

## Materials and methods

### Functional genome analysis

The functional genome analysis of PhiMa05 (GenBank accession number: MW495066.1) was conducted using a combination of NCBI Protein database [15] mining, RefSeq BLASTP [16] searches and, when RPs were detected, InterProScan [17] runs and Foldseek [18] queries against experimentally determined structures in the PDB [19] in order to confirm their annotations using multiple tools. Unless otherwise noted, all tools were executed with default parameters. BLASTP hits with *E*-values <0.001 were considered significant, as were Foldseek hits with *E*-values <0.001 and true-positive probabilities of 1 [20, 21]. Structural modeling of PhiMa05 RPs for Foldseek queries was conducted using AlphaFold 3 [22] (AF3), with the exception of bS1. The overall fold of AlphaFold models with predicted template modeling (pTM) scores >0.5 is usually considered similar to that of the true structure [21]. Since AF3 failed to produce a satisfactory model for bS1 (pTM < 0.5), D-I-TASSER [23] was used instead. To ensure optimal modeling accuracy, the following advanced options were activated in D-I-TASSER: using large IMG/JGI metagenomic database to build MSA, predict protein function based on structure model, using AlphaFold2 distances, and using domain partition and assembly module. As with AF3 pTM, an estimated template modeling (eTM) score >0.5 has been used as threshold to denote correctly folded D-I-TASSER models [23]. Prior to Foldseek searches, the predicted local-distance difference test scores of the AF3 models were visually inspected in the native environment of the AlphaFold server and can be found in Fig. S2. The structural alignments generated by Foldseek were visualized using UCSF ChimeraX 1.10 [24]. Additionally, InterProScan runs and AF3-Foldseek queries against experimentally determined structures in the PDB were also applied to the hypothetical proteins of PhiMa05.

### Phylogenetic analyses

We reconstructed both broad coverage and *Vampirovibrionia*-specific phylogenies for PhiMa05 RPs. To compute broad coverage trees, the top 20 RefSeq BLASTP hits of PhiMa05 RPs were aligned using Muscle 5.3 [25] and visualized in SeaView [26]. Blocks of conserved sites were selected via Gblocks [27], and maximum likelihood phylogenies were reconstructed using PhyML 3.1 [28] with the LG amino acid substitution model [29] and 100 bootstrap replicates. Since the ribosome biogenesis tree was dominated by *Bacillus* spp., we retained one *Bacillus* homolog (*B. altitudinis*, the most similar to PhiMa05) and conducted a second BLASTP search excluding the *Bacillus* genus (NCBI taxonomy ID: 1386) to compute a tree with wider taxonomic coverage. Subsequently, *Vampirovibrionia*-specific phylogenies were constructed using the top 20 *Vampirovibrionia* (1798710) and viral (10239) BLASTP hits in the NCBI non-redundant protein sequences database. *Vampirovibrio chlorellavorus* Vc_AZ_2 (see section 2.3) and *V. chlorellavorus* Vc_UKR (JGI IMG/MER accession number: 2600254900) RPs were also added to these trees.

In the case of the portal protein alignment, the use of Gblocks with default parameters yielded an insufficient number of positions, resulting in a tree with low bootstrap support for some branches. Therefore, Gblocks was executed with the following parameters for a less stringent selection to increase the robustness of the portal protein tree: allow smaller final blocks, allow gap positions within the final blocks, and allow less strict flanking positions.

### Multiple genome alignments

A multiple genome alignment between PhiMa05, *V. chlorellavorus* Vc_AZ_1 (contig QFWH01000002.1), and *V. chlorellavorus* Vc_AZ_2 (contig QFWI01000011.1) was performed using progressiveMauve [30]. Since the *V. chlorellavorus* Vc_AZ_2 genome was not annotated in NCBI, Prokka 1.14.5 [31] was used to annotate it before alignment. After examining the alignment, we observed that the only copies of certain RP-coding genes in Vc_AZ_1 and Vc_AZ_2 were located within PhiMa05-like prophages (see section 3.2). We confirmed their absence elsewhere in the genomes by conducting BLASTP searches with these RPs against the proteomes of Vc_AZ_1 and Vc_AZ_2.

In order to identify RPs located within PhiMa05-like prophages in *Vampirovibrionia* genomes, multiple genome alignments between PhiMa05 and each *Vampirovibrionia* genome were performed (Fig. S3). The RPs located within PhiMa05-like prophages were classified accordingly, while the RPs located outside of PhiMa05-like prophages were not identified as PhiMa05-like prophage RPs.

## Results

### Functional genome analysis of PhiMa05

**Figure 1:**
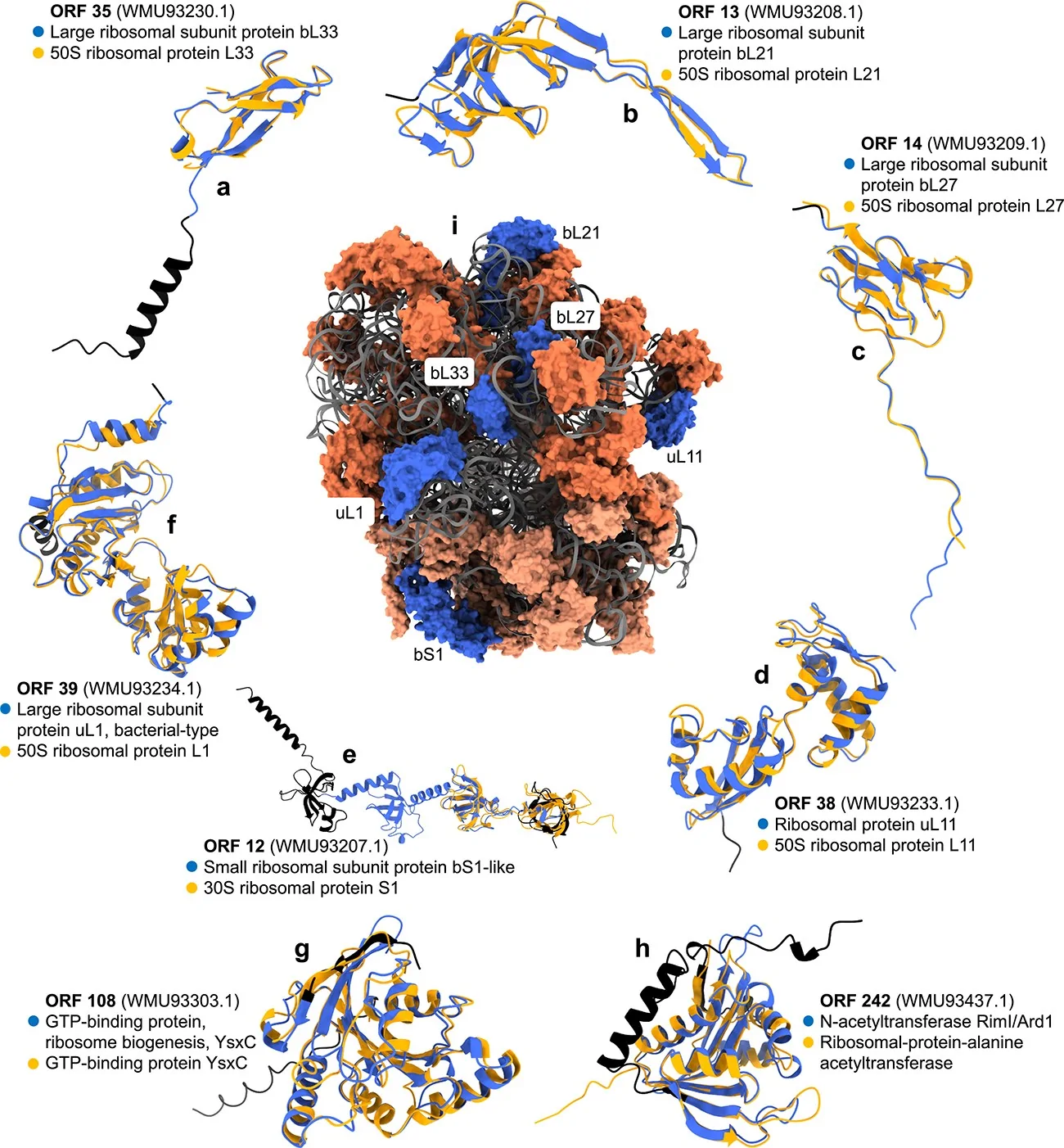
RPs encoded in the genome of PhiMa05 and their locations in a bacterial ribosome. (a) bL33, (b) bL21, (c) bL27, (d) uL11, (e) bS1, (f) uL1, (g) ribosome biogenesis GTP-binding YihA/YsxC protein, and (h) RP S18-alanine N-acetyltransferase. (i) Shows a bacterial ribosome (PDB ID: 6BU8; from *Escherichia coli*) with the RPs encoded in the PhiMa05 genome colored in blue, the remaining large subunit RPs in coral, the remaining small subunit RPs in light salmon, and the RNAs in gray. For (a-h), the structural models of PhiMa05 proteins are colored in black, except for the regions corresponding to their InterPro families, which are colored in royal blue. The top Foldseek PDB hits are colored in orange and structurally aligned to the PhiMa05 structural models. The InterPro family names of the PhiMa05 proteins are displayed next to closed blue circles, while the names of their top Foldseek PDB hits are listed next to closed orange circles. ORF numbers of PhiMa05 proteins are highlighted in bold, and their GenBank accession numbers are indicated in parentheses. Note that the different panels are not to scale. See Table S1 for BLASTP *E*-values; Table S2 for Foldseek *E*-values and InterPro family IDs; and Fig. S2 for the predicted local-distance difference test scores of the structural models.

Our functional genome analysis uncovered six RPs in the PhiMa05 genome, namely, bS1 (ORF 12), bL21 (ORF 13), bL27 (ORF 14), bL33 (ORF 35), uL11 (ORF 38), and uL1 (ORF 39), as well as a ribosome biogenesis GTP-binding YihA/YsxC protein (ORF 108), and an RP S18-alanine N-acetyltransferase (ORF 242; Fig. 1, Table S1). Specifically, NCBI Protein database mining rescued five RPs annotated in GenBank (bS1, bL21, bL27, uL11, and uL1); while BLASTP, InterProScan, and Foldseek queries (Table S2) confirmed those five and revealed three more: bL33, the RP S18-alanine N-acetyltransferase, and the ribosome biogenesis protein (previously annotated only as a “GTP-binding protein”). It is worth noting that, except for uL11, the RPs encoded by PhiMa05 are located moderately close to one another in a prokaryotic ribosome (Fig. 1i). A possible explanation for this observation is that such proteins might interact during the biogenesis of the ribosome or the protein synthesis process. The viral genome also contains a few tRNA genes (tRNA-Trp, EMOOHJMP_00036; tRNA-Asn, EMOOHJMP_00158; and tRNA-Arg, EMOOHJMP_00211; mined from NCBI). To the best of our knowledge, this makes PhiMa05 the first cultivated cyanophage reported to encode RPs, as well as the virus with the most comprehensive RP-coding set of the known virosphere.

In order to further explore the genomic dark matter of PhiMa05, we also applied InterProScan runs and Foldseek queries to the hypothetical proteins of the virus (Table S3). A hypothetical protein (ORF 225) got an InterProScan hit to the RP uL14 family. However, this result was not consistent among tools. Therefore, we decided not to annotate ORF 225 as an RP in our study. Another highlight was ORF 199, which shares a genomic cluster with PhiMa05 structural proteins. Accordingly, ORF 199 was found to be a structural homolog of the packaged DNA stabilization protein of Salmonella phage P22, a temperate bacteriophage.

### Evolutionary analyses

**Figure 2:**
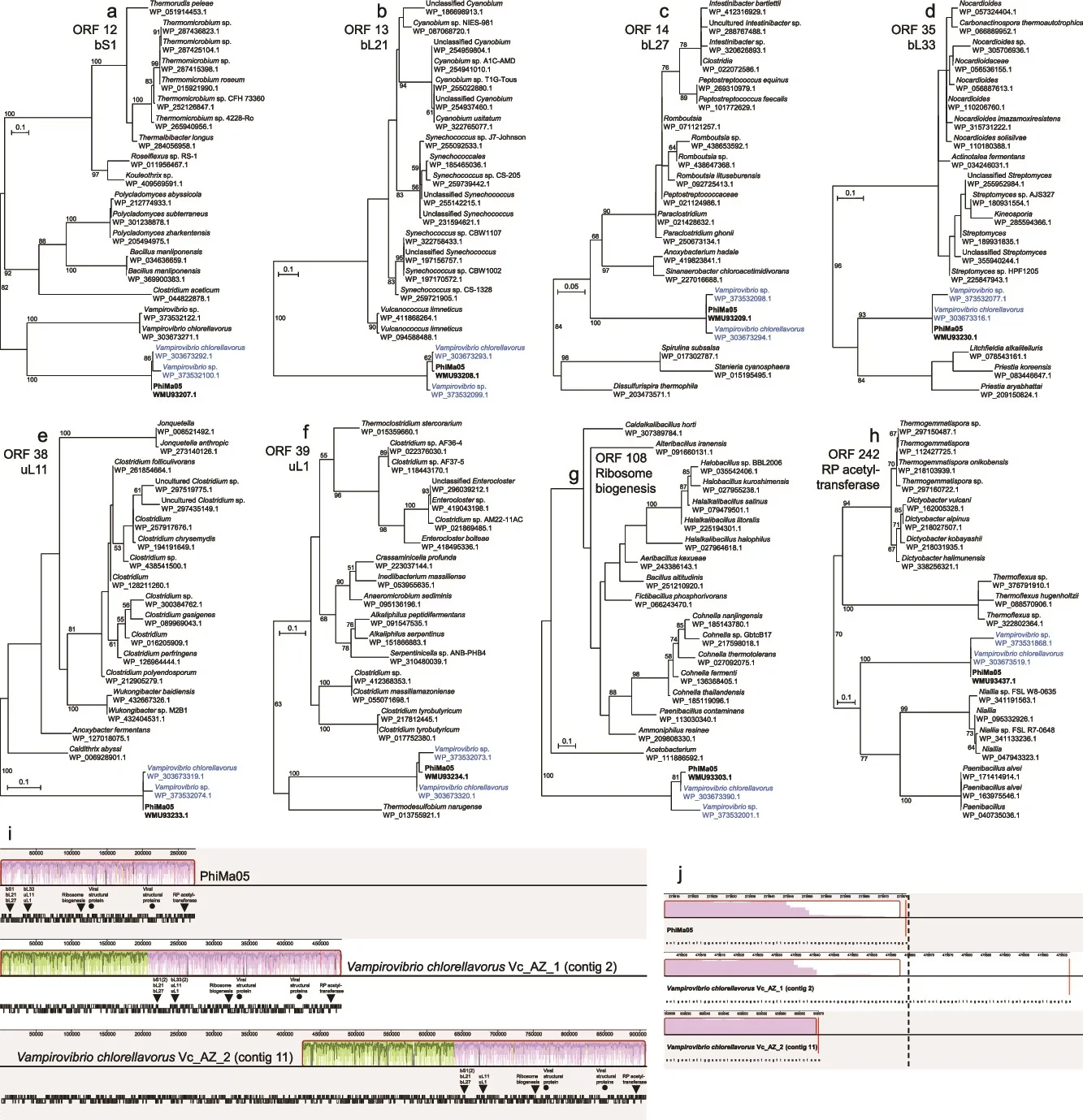
Broad coverage phylogenies of PhiMa05 RPs and synteny comparison between the PhiMa05 genome and *V. chlorellavorus* prophages. (a) bS1, (b) bL21, (c) bL27, (d) bL33, (e) uL11, (f) uL1, (g) ribosome biogenesis GTP-binding YihA/YsxC protein, and (h) RP S18-alanine N-acetyltransferase maximum likelihood phylogenetic trees. The PhiMa05 ORF numbers and protein names are indicated next to the trees. Support values >50 (out of 100 bootstrap replicates) are shown at the nodes. The scale bars indicate the number of amino acid substitutions per site. RefSeq accession numbers are included below the species names. PhiMa05 proteins are highlighted in bold, and PhiMa05-like prophage proteins are colored in blue. (i) Synteny analysis between PhiMa05 (top), *V. chlorellavorus* Vc_AZ_1 (middle), and *V. chlorellavorus* Vc_AZ_2 genomes (bottom). The locally colinear blocks (LCBs) corresponding to the PhiMa05 genome are colored in mauve, while the remaining syntenic regions between the Vc_AZ_1 and Vc_AZ_2 contigs are colored in green. The plots within the LCBs display sequence similarity values as a range with the mean value darkened; the height of the similarity profile indicates the level of sequence conservation over a region, calculated as inversely proportional to the average column entropy of the alignment. The coding sequences are displayed as black and white bars below each genome; PhiMa05 RPs and structural proteins (including tail, adaptor, capsid, and portal proteins) are indicated using closed black triangles and circles, respectively. (j) Close-up view of the right ends of the PhiMa05 genome and *V. chlorellavorus* prophage-containing contigs. Red lines mark the end of the contigs, while the black dotted line highlights the end of the PhiMa05 genome.

The RPs of PhiMa05 raise questions regarding their evolutionary trajectories. In all of our phylogenetic analyses, the RPs from PhiMa05 are strongly supported as being closely related to *Vampirovibrio* RPs (Fig. 2a-h). For context, *Vampirovibrio* is a genus of predatory cyanobacteria that belongs to a basal non-photosynthetic class of the phylum *Cyanobacteriota*, the *Vampirovibrionia* (previously *Melainabacteria*) [32]. The ecology of *V. chlorellavorus* is quite remarkable, as it is an obligate predator of the green microalga *Chlorella* [32, 33]. Interestingly, the comparative genomic analyses implemented here reveal that there is a highly conserved PhiMa05-like prophage in the genomes of *V. chlorellavorus* strains Vc_AZ_1 (GenBank accession number: GCA_003149375.1) and Vc_AZ_2 (Fig. 2i; GCA_003149345.1), which were isolated from outdoor experimental algal cultivation ponds in Arizona [33]. The PhiMa05-like prophages in Vc_AZ_1 and Vc_AZ_2 are flanked on the left side by host genomic regions. Although the Vc_AZ_2 contig ends on the right side before the PhiMa05-like prophage does, the right end of the Vc_AZ_1 prophage is also flanked by a host region (Fig. 2j), providing evidence of integration on both sides of the viral genome in Vc_AZ_1.

PhiMa05 and the Vc_AZ_1 and Vc_AZ_2 prophages do not encode any recognizable integrases. This is in line with previous studies on the *Saffermanviridae* family of temperate cyanophages, which is the viral lineage most closely related to PhiMa05 (see section 1 and Fig. S1). Viruses in the *Saffermanviridae* are able to lysogenize their freshwater hosts despite the lack of detectable integrases [14].

**Figure 3:**
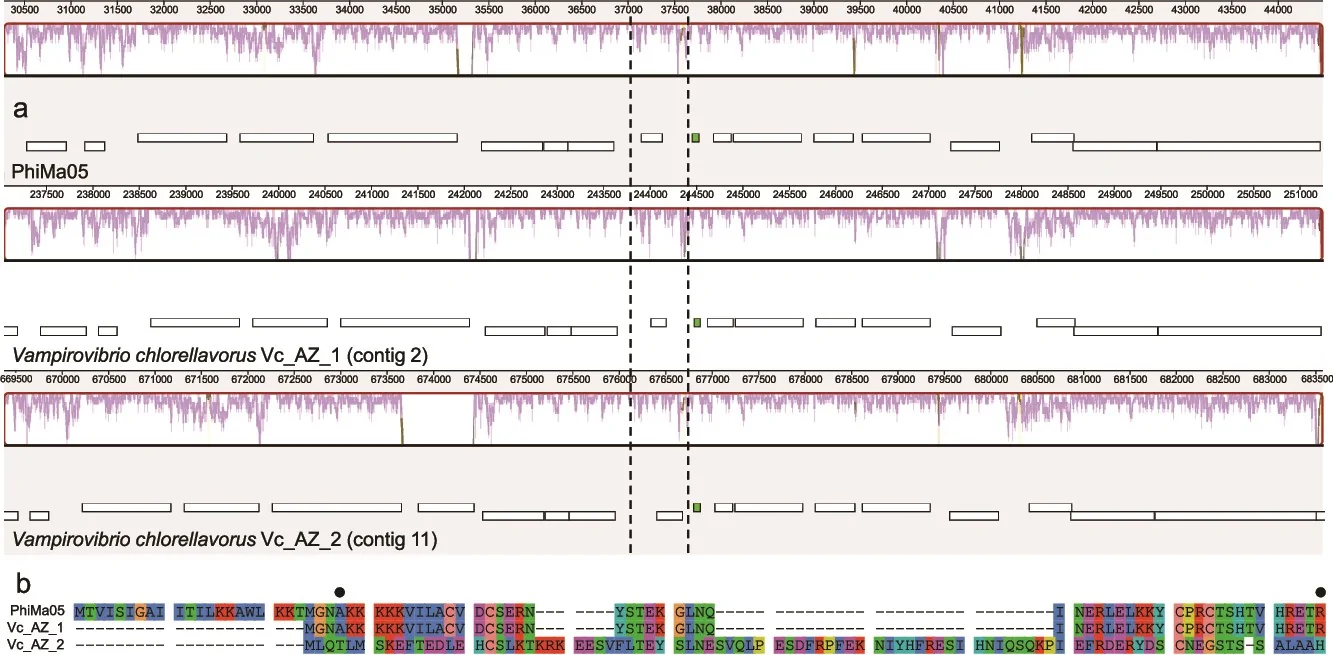
Alignment of RP bL33. (a) Close-up view of bL33 ORFs in the PhiMa05 genome (top), *V. chlorellavorus* Vc_AZ_1 (middle), and *V. chlorellavorus* Vc_AZ_2 (bottom) PhiMa05-like prophages. Black dotted lines enclose bL33 ORFs in PhiMa05 and Vc_AZ_1, and ORF 651 in Vc_AZ_2. The gene colored in green encodes tRNA-Trp. (b) Multiple sequence alignment of PhiMa05 bL33 (GenBank accession number: WMU93230.1), *V. chlorellavorus* Vc_AZ_1 prophage bL33 (WP_303673316.1), and *V. chlorellavorus* Vc_AZ_2 ORF 651 (HHJJEAIM_00651). Closed circles denote the intervals of amino acids in PhiMa05 and Vc_AZ_1 proteins corresponding to the bL33 family. No hits were detected when querying ORF 651 in BLASTP and InterProScan.

Although the degree of conservation between PhiMa05 and *V. chlorellavorus* prophages is high, they are not perfectly conserved. As can be observed in the multiple genome alignment in Fig. 2i, the sequence conservation is never absolute throughout the PhiMa05-like prophages. A few spots are particularly divergent, as evidenced by the pronounced valleys present in the sequence similarity plots. RP bL33 in the Vc_AZ_2 prophage provides a specific example of divergence and pseudogenization. There is an ORF (HHJJEAIM_00651) where bL33 used to be in the Vc_AZ_2 prophage, but its coding sequence has mutated to such an extent as to bear no detectable homology and almost no sequence similarity to bL33 proteins (Fig. 3). Indeed, when querying ORF 651, no significant similarity can be detected using BLASTP, and InterProScan fails to identify it as part of any protein family. These are signs of pseudogenization and divergence between PhiMa05 and *V. chlorellavorus* prophages, which comprise another line of evidence supporting the integration of the viral genome.

**Figure 4:**
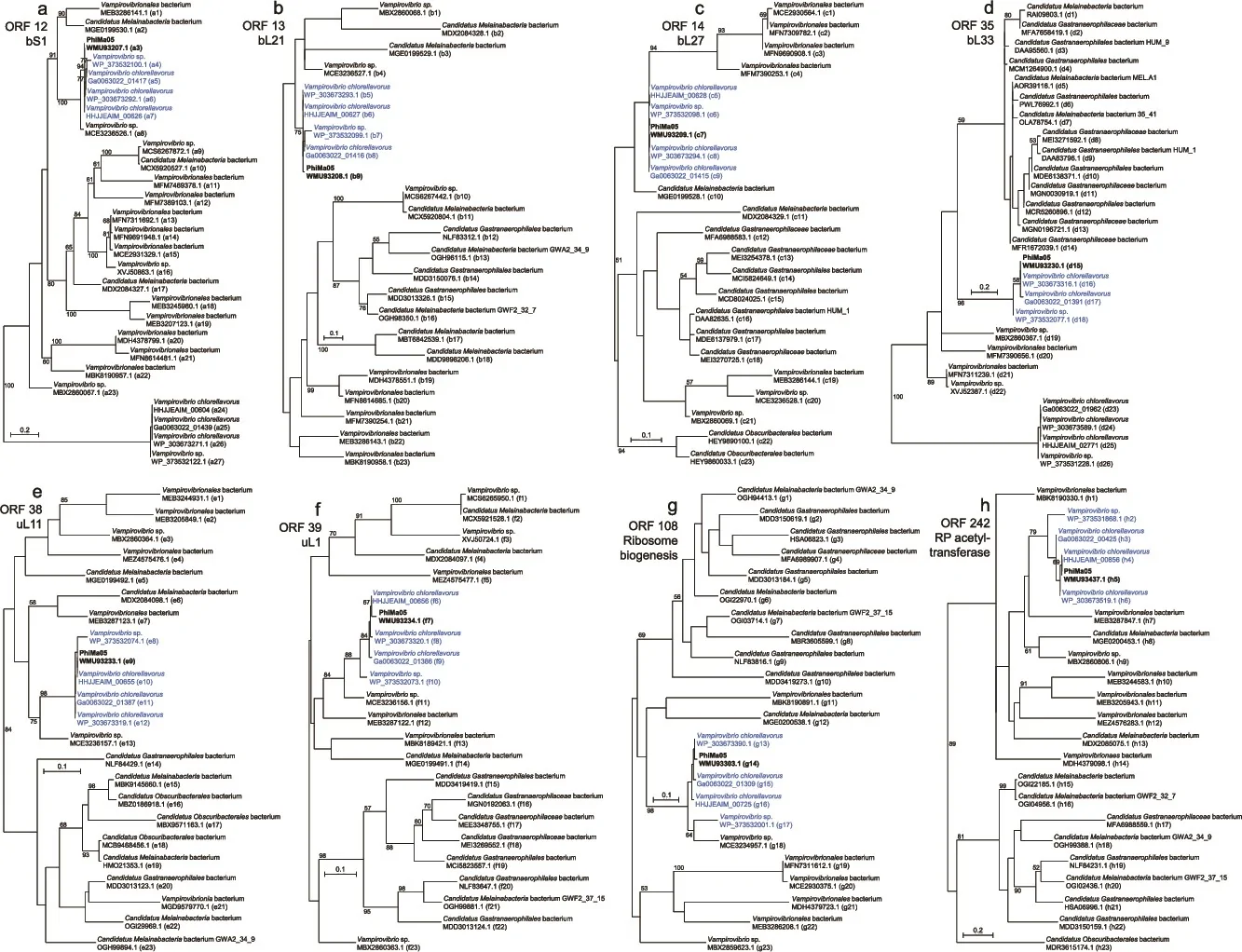
*Vampirovibrionia*-specific phylogenies of PhiMa05 RPs. (a) bS1, (b) bL21, (c) bL27, (d) bL33, (e) uL11, (f) uL1, (g) ribosome biogenesis GTP-binding YihA/YsxC protein, and (h) RP S18-alanine N-acetyltransferase maximum likelihood phylogenetic trees. The PhiMa05 ORF numbers and protein names are indicated next to the trees. Support values >50 (out of 100 bootstrap replicates) are shown at the nodes. The scale bars indicate the number of amino acid substitutions per site. Accession numbers are included below the species names. PhiMa05 proteins are highlighted in bold, and PhiMa05-like prophage proteins are colored in blue. The parentheses following the accession numbers enclose the identifiers of each RP used to indicate their locations in the genome alignments in Fig. S3.

Most *Vampirovibrio* RPs in our broad coverage trees belonged to PhiMa05-like prophages (Fig. 2a-h). As such, it was not clear whether PhiMa05 acquired its RP-coding genes from a *Vampirovibrio*-like host. Therefore, the reconstruction of higher resolution *Vampirovibrionia*-specific RP phylogenies followed. In these trees, PhiMa05 and PhiMa05-like prophages clustered together and further grouped with *Vampirovibrio* RPs (Fig. 4). This suggests that the PhiMa05 RP-coding genes may have been acquired by an ancestor of the virus from a *Vampirovibrio*-like host.

We observed that uL1, uL11, bL21, bL27, the ribosome biogenesis GTP-binding YihA/YsxC, and the RP S18-alanine N-acetyltransferase of the PhiMa05-like prophages were the only copies of these proteins detected in the near-complete Vc_AZ_1 and Vc_AZ_2 host genomes. Because Vc_AZ_1 and Vc_AZ_2 are contig-level assemblies, we cannot rule out the possibility that the aforementioned RPs are located in contig gaps. However, this is unlikely because the Vc_AZ_1 and Vc_AZ_2 genomes are near complete, and the fraction of potentially missed genes in contig gaps is minimal [32, 33]. Furthermore, Vc_AZ_1 and Vc_AZ_2 lack the same six RPs beyond the copies present in the PhiMa05-like prophages, making it highly improbable that the same six proteins are independently present in gap regions in both strains. Moreover, there is an ORF coding for bL33, which is the only RP-coding gene that has pseudogenized in the PhiMa05-like prophages, located elsewhere in the Vc_AZ_2 genome (HHJJEAIM_02771).

## Discussion

In order to interpret the biological implications of RP acquisition in PhiMa05, we begin by examining the functional roles of the RPs encoded by the virus. Protein bS1 enables translation initiation by binding mRNA, recruiting it to the 30S ribosomal subunit, and facilitating the accommodation of the start codon within the decoding center [34, 35]. In addition, bS1 has been found to be involved in *trans*-translation, a process that rescues stalled ribosomes [36], as well as in regulating its own expression and that of other ribosomal genes [37, 38]. Furthermore, bS1 plays a role in the replication cycle of several bacteriophages. In T4, the host bS1 participates in replication through the activation of the phage endoribonuclease RegB, resulting in the degradation of early viral mRNAs [39]. In bacteriophage Qβ, the cellular bS1 comprises a subunit of the viral replicase complex that acts as a replication initiator and terminator factor [40, 41]. RP bS1 interacts with the temperate phage λ recombination protein β, thus taking part in the recombination process during infection [42, 43]. Beyond bS1, which is frequently lost in bacteria [44], none of the other RPs encoded in PhiMa05 has been reported to exhibit any particular conservation trend.

Together with 23S rRNA, uL1 forms the L1 stalk, a flexible structural element located near the E (exit) site of the ribosome [45]. The L1 stalk mediates the release of tRNA from the E site by interacting with deacylated tRNA during translocation [46]. Similar to bS1, uL1 also works as a repressor that regulates the expression of several RPs, including its own [47]. Research on bL33 is limited, but it has been found to contact tRNAs directly in the E site as well [48]. RP uL11 is part of the L7/L12 stalk on the bacterial 50S subunit, where it takes part in the recruitment and stabilization of GTP-binding translation factors and promotes GTP hydrolysis during translation [38, 49]. Protein bL27 is implicated in 50S subunit assembly and contributes to the peptidyl-transferase reaction by positioning the acceptor end of aminoacyl-tRNAs at the peptidyl transferase center [50, 51]. In contrast, the function of bL21 remains obscure. Overall, it has been experimentally demonstrated that viral RPs can replace cellular homologs in host ribosomes [4]. Consequently, PhiMa05 RPs may replace their *Microcystis* counterparts during infection.

RP S18-alanine N-acetyltransferase (RimI), as its name suggests, acetylates the N-terminal alanine of bS18 [52–54]. Although the function of bS18 is unknown, it is essential for ribosomal function and cell survival [36]. Not surprisingly, the Vc_AZ_1 and Vc_AZ_2 host genomes encode bS18 in their genomes (WP_303673965.1 and HHJJEAIM_01006, respectively). RimI also catalyzes the acetylation of several other proteins [55–57], including the elongation factor Tu (EF-Tu) [58]. EF-Tu, one of the most abundant and crucial bacterial proteins, is responsible for delivering aminoacyl-tRNAs to the ribosome’s aminoacyl site during protein synthesis [59, 60]. In viruses, EF-Tu plays a role in T4 head assembly and is a subunit of the Qβ replicase [61, 62]. EF-Tu protein-coding genes are present in one copy in the Vc_AZ_1 genome (WP_303672637.1) and in two copies in Vc_AZ_2 (HHJJEAIM_00211 and HHJJEAIM_01385).

The GTP-binding protein YsxC is essential for the biogenesis of the ribosome and the survival of the bacterial cell [63–66]. Its mechanism of action as a placeholder for the essential uL2 protein during ribosome assembly has been recently elucidated. During the biogenesis of the large ribosomal subunit, YsxC binds to the location that will be subsequently occupied by uL2, where it contributes to the folding of several rRNA helices forming the uL2 binding site [67]. Following the release of YsxC, uL2 binds to the site previously occupied by its placeholder, after which the remaining rRNA helices stabilize the uL2 fold, and the region transitions into its mature conformation [67]. As expected, Vc_AZ_1 and Vc_AZ_2 encode bL2 (WP_303672632.1 and HHJJEAIM_00216, respectively).

As described in section 3.2, the genes coding for uL1, uL11, bL21, bL27, RimI, and YsxC in the PhiMa05-like prophages are the only copies detected in the near-complete Vc_AZ_1 and Vc_AZ_2 host genomes. Given the functions of these RPs and considering that the fraction of potentially missed genes in contig gaps in Vc_AZ_1 and Vc_AZ_2 is minimal [32, 33], we hypothesize that both cellular organisms may depend on the PhiMa05-like prophage for ribosome biogenesis, protein synthesis, and hence life itself. Indeed, there are several instances of organisms that rely on integrated viruses or endogenous viral genes to perform essential functions. One such case is mammalian placentation. The mouse *syncytin-A* gene of retroviral origin is essential for differentiation of trophoblasts and morphogenesis of syncytiotrophoblasts during the development of the placenta [68]. Without this viral gene, mice embryos die during gestation [68]. As for bacteriophages, the mitochondrial gamma DNA polymerase is probably of phage origin [69]; and almost half of sequenced bacterial genomes contain prophages, many of which encode adaptive traits [70].

**Figure 5:**
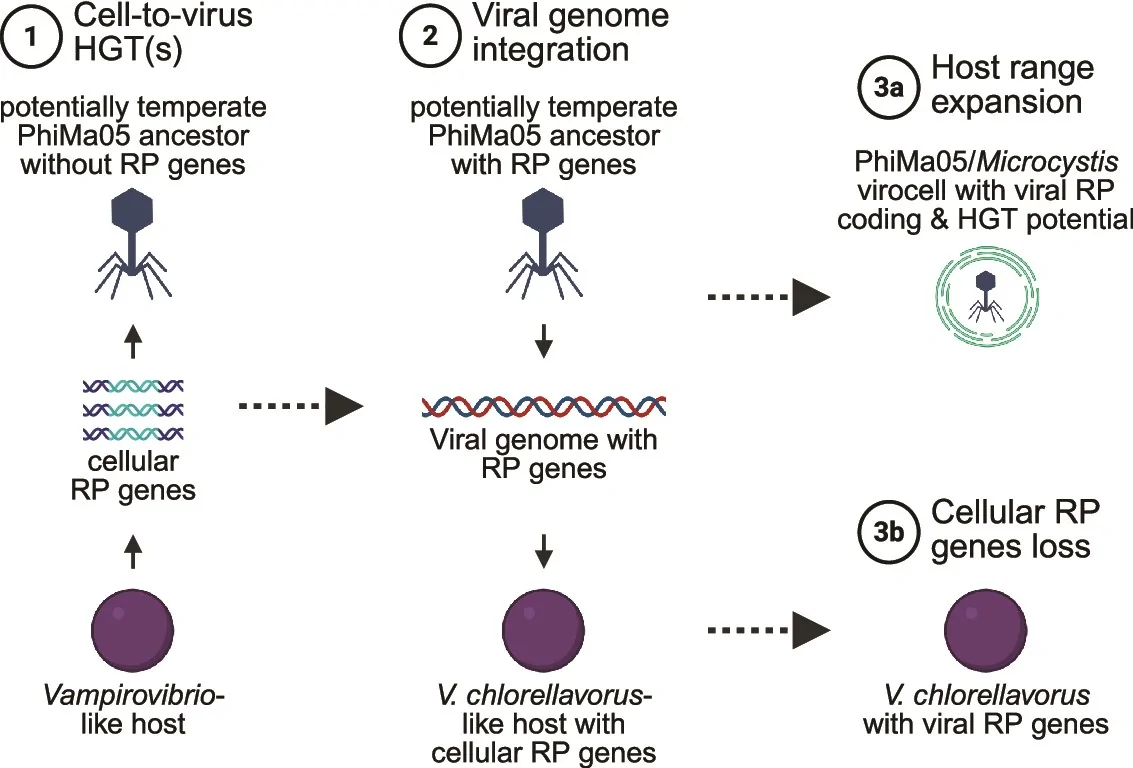
Eco-evolutionary model for the causes and consequences of PhiMa05 RP-coding genes. In step 1, an ancestor of PhiMa05 horizontally acquired cellular RP genes from a *Vampirovibrio*-like host. In step 2, there was a viral genome integration event in which a potentially temperate ancestor of PhiMa05 integrated its genome with viral RP genes into a *V. chlorellavorus*-like host that probably had its own cellular set of RP genes. In step 3b (the cellular side), certain *V. chlorellavorus* lost the cellular copies of four RP genes, an RP acetyltransferase, and a ribosome biogenesis protein, resulting in *V. chlorellavorus* strains with only viral versions of these genes. In step 3a (the viral side), the PhiMa05 ancestor experienced a host range expansion or shift, leading to the modern PhiMa05/*Microcystis* virocell with viral RP coding and horizontal gene transfer (HGT) potential from the viral world into this new cellular genus. Created in BioRender.

Based on the results presented here, we propose the following eco-evolutionary scenario (Fig. 5). First, a potentially temperate ancestor of PhiMa05 horizontally acquired RP-coding genes from a *Vampirovibrio*-like host. Second, the PhiMa05 ancestor integrated its genome in a *V. chlorellavorus* Vc_AZ_1/2-like host that probably carried its own cellular set of RP-coding genes. Third, the Vc_AZ_1/2-like host lost the cellular copies of the genes coding for uL1, uL11, bL21, bL27, the ribosome biogenesis, and the RP acetyltransferase proteins (i.e. the viral versions of these genes were selected for over their cellular counterparts). As a result, the only genes for four RPs, a ribosome biogenesis protein, and an RP acetyltransferase encoded in the genomes of *V. chlorellavorus* Vc_AZ_1 and Vc_AZ_2 are of viral (prophage) origin. Whether these prophages are currently inducible remains unclear and should be experimentally and ecologically investigated in future studies. Over the course of evolution, the PhiMa05 ancestor likely experienced an inter-class host range expansion or shift, leading to the current *Microcystis*-infecting PhiMa05 containing RP-coding genes. Given that jumbo phages have wider host ranges than smaller phages [71], the close association of *Vampirovibrionales* with toxic *Microcystis* blooms [72], and the existence of *Microcystis* phages with exceedingly broad host ranges [73], such host expansion or shift would indeed be possible from an ecological framework. Overall, our findings provide evidence for the existence of viruses with particularly enriched sets of RP-coding genes and indicate that, in some cases, such viral genes have been transferred to cells, potentially becoming essential for the survival of the host.

Viruses and cells were redefined in 2008 as capsid- and ribosome-encoding organisms, respectively [1]. Several other approaches to the question have been proposed as well [74, 75], including the classic definition of viruses as obligate intracellular parasites. The virocell concept, positing that the infected cell is the actual organism [76], is another insightful development that provides a framework for studying viruses within systems, information [77], and dynamic kinetic stability theories [78, 79]. Certainly, biological systems are replicators that interact and coevolve (and profoundly affect their surroundings) regardless of their viral or cellular nature [80]. As this is a viral RP study, we frame our discussion within a capsid- and ribosome-encoding definition of biological entities and argue that the discoveries presented here support the continuous nature of virus-host systems.

## Supplementary Material

Supplementary_Material_wrag084

## Data Availability

The structural models of PhiMa05 proteins and the genome annotation files of *V. chlorellavorus* Vc_AZ_2 have been deposited in Figshare [81]. The rest of the data is included in the manuscript, supplementary material, or available in public databases. Tables are also provided as spreadsheets in Supplementary Data 1. Accession numbers can be found throughout the text.
