# Supplementary material for: A jumbo cyanophage encodes the most comprehensive ribosomal protein set in the known virosphere
Source: ISME J. 2026 Apr 10;20(1):wrag084. doi: 10.1093/ismejo/wrag084 (PMC13157829; doi:10.1093/ismejo/wrag084)
Supplement: Supplementary_Material_wrag084 [file supplementary_material_wrag084.zip › Meza-Padilla_supplementary_wrag084.pdf]

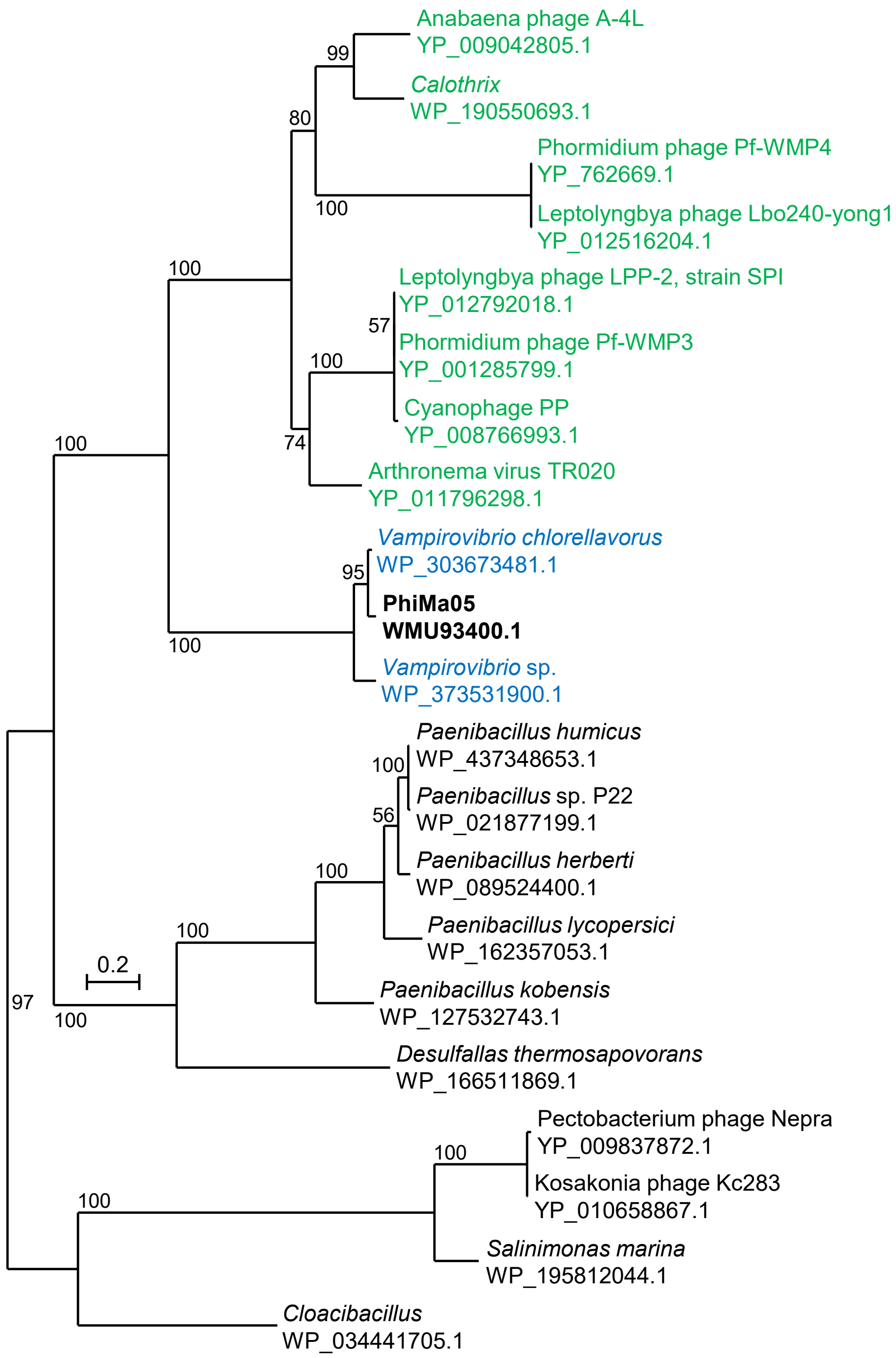

Fig. S1. Maximum likelihood phylogenetic tree of PhiMa05 portal protein. Support values > 50 (out of 100 bootstrap replicates) are shown at the nodes. The scale bar indicates the number of amino acid substitutions per site. RefSeq accession numbers are included below the species

names. PhiMa05 is highlighted in bold, and PhiMa05-like prophages are coloured in blue. Viruses (a prophage in the case of *Calothrix*) belonging to the *Saffermanviridae* family are depicted in green.

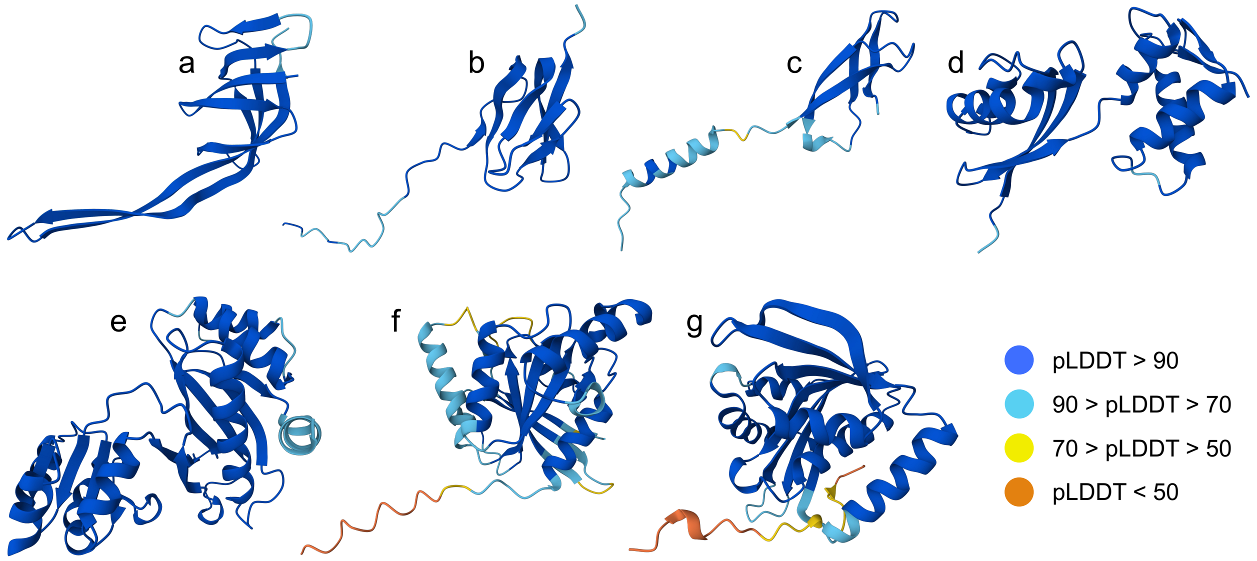

Fig. S2. Predicted local-distance difference test (pLDDT) scores of the AlphaFold 3 structural models of PhiMa05 ribosomal proteins (RPs). (a) bL21, (b) bL27, (c) bL33, (d) uL11, (e) uL1, (f) ribosome biogenesis GTP-binding YihA/YsxC protein, and (g) RP S18-alanine N-acetyltransferase. The different panels are not to scale. bS1 is not shown because D-I-TASSER models do not include pLDDT scores. However, the estimated template modelling score of the bS1 structural model can be found in **Table S2**.

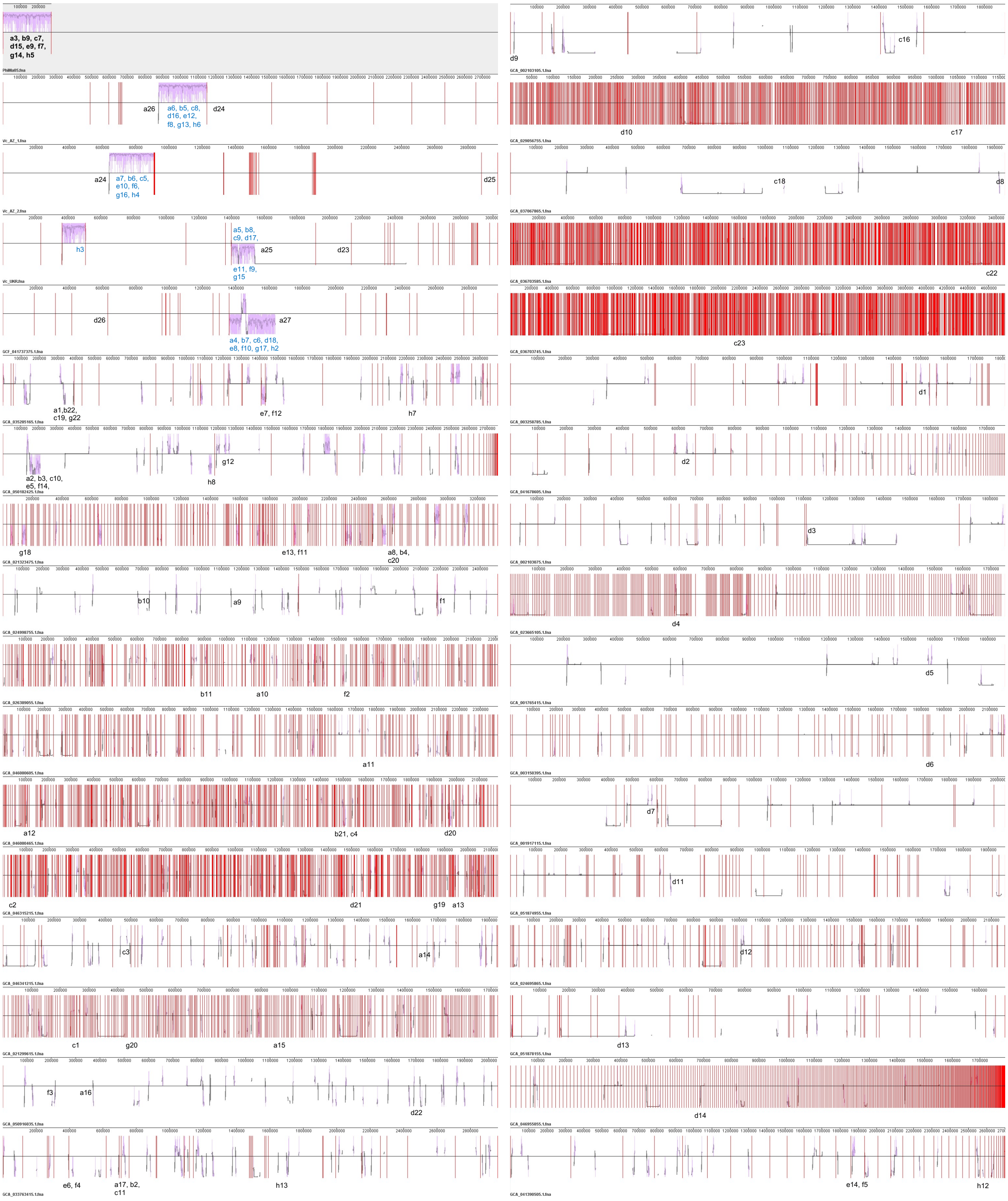

Figure continues on next page.

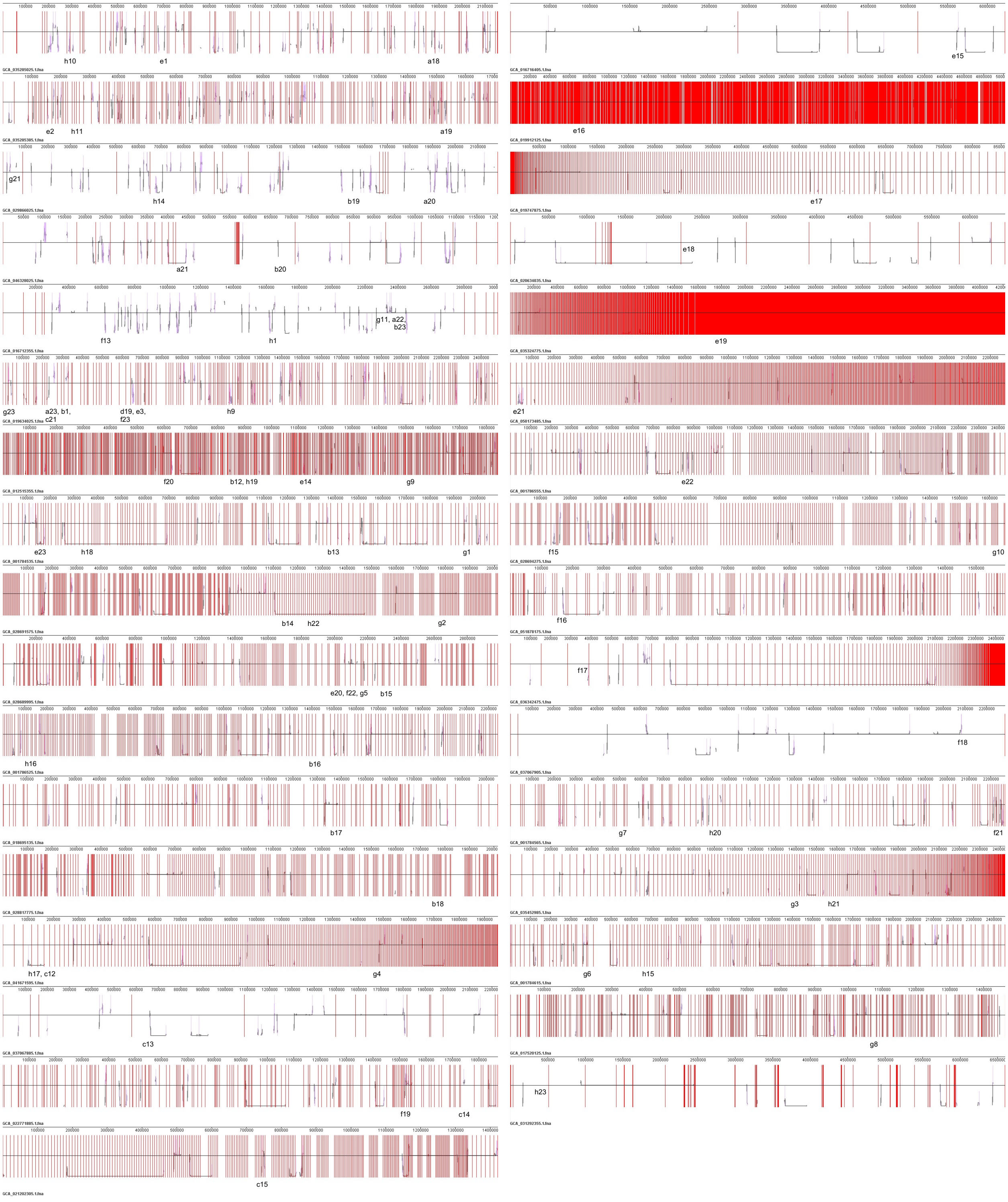

Fig. S3. Genome alignments between PhiMa05 and *Vampirovibrionia* strains from **Fig. 4**. The locally colinear blocks (LCBs) corresponding to the PhiMa05 genome are coloured in mauve. The plots within the LCBs display sequence similarity values as a range with the mean value

darkened; the height of the similarity profile indicates the level of sequence conservation over a region, calculated as inversely proportional to the average column entropy of the alignment. Red lines mark the end of the contigs. PhiMa05 proteins are highlighted in bold, and PhiMa05-like prophage proteins are coloured in blue. Note that the PhiMa05-like prophage in Vc_UKR is split across two contigs. The key to the protein identifiers used in this figure can be found in **Fig. 4**. Each protein entry of the trees in **Fig. 4** has a unique identifier indicated in parentheses after the accession number. For example, the identifier 'a12' refers to ribosomal protein bS1 (MFM7389103.1) of *Vampirovibrionales* bacterium, and 'b9' refers to ribosomal protein bL21 (WMU93208.1) of PhiMa05. The accession numbers of the genomes are displayed in the bottom-left corners of the progressiveMauve plots.

Table S1. Annotations and homologs of PhiMa05 open reading frames. Ribosomal proteins are underlined.

ORF	GenBank accession	Naknaen et al.[11] annotation[a] (2021 paper)	GenBank annotation (2023 revision)	RefSeq homolog[b] (this study)	Organism (this study)	BLASTP *E*-value (this study)
1	WMU93196.1	4-hydroxy-3-methylbut-2-enyl diphosphate synthase	4-hydroxy-3-methylbut-2-en-1-yl diphosphate synthase	Flavodoxin-dependent (E)-4-hydroxy-3-methylbut-2-enyl-diphosphate synthase	Unclassified *Clostridium*	1e-129
2	WMU93197.1	N.a.	HP	2 HPs	*Vampirovibrio* spp.	≤ 2e-45
3	WMU93198.1	N.a.	HP	Radical SAM protein	*V. chlorellavorus*	0
4	WMU93199.1	N.a.	Cyclic dehypoxanthine futalosine synthase	Cyclic dehypoxanthinyl futalosine synthase	*V. chlorellavorus*	0
5	WMU93200.1	N.a.	HP	2 HPs	*Vampirovibrio* spp.	≤ 5e-96
6	WMU93201.1	N.a.	HP	2 HPs	*Vampirovibrio* spp.	≤ 1e-38
7	WMU93202.1	Tryptophanyl-tRNA synthetase	Tryptophan--tRNA ligase	Tryptophan--tRNA ligase	*V. chlorellavorus*	0
8	WMU93203.1	N.a.	HP	2 HPs	*Vampirovibrio* spp.	≤ 2e-104
9	WMU93204.1	N.a.	HP	Prepilin peptidase	*V. chlorellavorus*	0
10	WMU93205.1	Putative RNA polymerase sigma subunit	RNA polymerase sigma factor	Sigma-70 family RNA polymerase sigma factor	*V. chlorellavorus*	0
11	WMU93206.1	N.a.	4-hydroxy-3-methylbut-2-enyl diphosphate reductase	4-hydroxy-3-methylbut-2-enyl diphosphate reductase	*V. chlorellavorus*	0
12	WMU93207.1	N.a.	30S ribosomal protein S1	30S ribosomal protein S1	*V. chlorellavorus*	0
13	WMU93208.1	N.a.	50S ribosomal protein L21	50S ribosomal protein L21	*V. chlorellavorus*	2e-69
14	WMU93209.1	N.a.	50S ribosomal protein L27	50S ribosomal protein L27	*V. chlorellavorus*	3e-53
15	WMU93210.1	N.a.	HP	2 HPs	*Vampirovibrio* spp.	≤ 4e-13
16	WMU93211.1	N.a.	Holliday junction ATP-dependent DNA helicase	Holliday junction branch migration protein RuvA	*V. chlorellavorus*	5e-142
17	WMU93212.1	N.a.	2-C-methyl-D-erythritol 4-phosphate cytidylyltransferase	2-C-methyl-D-erythritol 4-phosphate cytidylyltransferase	*V. chlorellavorus*	7e-103
18	WMU93213.1	N.a.	Thiamine-monophosphate kinase	Thiamine-phosphate kinase	*V. chlorellavorus*	0
19	WMU93214.1	N.a.	HP	2 HPs	*Vampirovibrio* spp.	≤ 2e-07
20	WMU93215.1	Aerotaxis sensor receptor protein	HP	Globin-coupled sensor protein	*V. chlorellavorus*	0
21	WMU93216.1	N.a.	HP	Type II secretion system F family protein	*V. chlorellavorus*	0
22	WMU93217.1	N.a.	HP	Type II secretion system F family protein	*V. chlorellavorus*	0
23	WMU93218.1	CpaF Flp pilus assembly protein, ATPase CpaF	HP	CpaF family protein	*V. chlorellavorus*	0
24	WMU93219.1	N.a.	Protein-glutamate methylesterase/ protein-glutamine glutaminase	AAA family ATPase	*V. chlorellavorus*	0
25	WMU93220.1	N.a.	Type 3 secretion system secretin	Type II and III secretion system protein family protein	*V. chlorellavorus*	0
26	WMU93221.1	N.a.	HP	Flp pilus assembly protein CpaB	*V. chlorellavorus*	0
27	WMU93222.1	N.a.	HP	TadE family protein	*V. chlorellavorus*	2e-83
28	WMU93223.1	N.a.	HP	3 HPs	*Vampirovibrio* spp.	≤ 7e-09

29	WMU93224.1	Thioredoxin reductase gp344	Thioredoxin reductase	Thioredoxin-disulfide reductase	*V. chlorellavorus*	0
30	WMU93225.1	N.a.	HP	2 HPs	*Vampirovibrio* spp.	≤ 4e-13
31	WMU93226.1	N.a.	3'3'-cGAMP-specific phosphodiesterase 3	HD-GYP domain-containing protein	*V. chlorellavorus*	0
32	WMU93227.1	N.a.	HP	DivIVA domain-containing protein	*Zarconia navalis*	2e-26
33	WMU93228.1	N.a.	HP	2 HPs	*Vampirovibrio* spp.	≤ 3e-52
34	WMU93229.1	CoaD Phosphopantetheine adenylyltransferase	Phosphopantetheine adenylyltransferase	Pantetheine-phosphate adenylyltransferase	*V. chlorellavorus*	8e-114
35	WMU93230.1	N.a.	HP	50S ribosomal protein L33	*V. chlorellavorus*	2e-31
36	WMU93231.1	N.a.	Protein translocase subunit	Preprotein translocase subunit SecE	*V. chlorellavorus*	2e-35
37	WMU93232.1	Transcription antitermination protein	Transcription termination/antitermination protein	Transcription termination/antitermination protein NusG	*Vampirovibrio* sp.	9e-151
38	WMU93233.1	N.a.	50S ribosomal protein L11	50S ribosomal protein L11	*V. chlorellavorus*	9e-98
39	WMU93234.1	N.a.	50S ribosomal protein L1	50S ribosomal protein L1	*V. chlorellavorus*	2e-169
40	WMU93235.1	AhpC Peroxiredoxin	Selenocysteine-containing peroxiredoxin	Peroxiredoxin	*V. chlorellavorus*	2e-126
41	WMU93236.1	N.a.	Thiol-disulfide oxidoreductase	Peroxiredoxin family protein	*V. chlorellavorus*	5e-68
42	WMU93237.1	N.a.	2-dehydro-3-deoxygluconokinase	Sugar kinase	*V. chlorellavorus*	0
43	WMU93238.1	N.a.	Dihydroxy-acid dehydratase	Dihydroxy-acid dehydratase	*V. chlorellavorus*	0
44	WMU93239.1	N.a.	KHG/KDPG aldolase	Bifunctional 4-hydroxy-2-oxoglutarate aldolase/2-dehydro-3-deoxy-phosphogluconate aldolase	*V. chlorellavorus*	5e-137
45	WMU93240.1	N.a.	Kynurenine formamidase	Cyclase family protein	*V. chlorellavorus*	4e-153
46	WMU93241.1	N.a.	HP	KGG domain-containing protein	*V. chlorellavorus*	8e-38
47	WMU93242.1	N.a.	Epoxyqueuosine reductase	tRNA epoxyqueuosine(34) reductase QueG	*V. chlorellavorus*	0
48	WMU93243.1	N.a.	Glucose-6-phosphate isomerase	Glucose-6-phosphate isomerase	*V. chlorellavorus*	0
49	WMU93244.1	Ferredoxin/ferredoxin--NADP reductase	Ferredoxin	4Fe-4S dicluster domain-containing protein	*V. chlorellavorus*	1e-52
50	WMU93245.1	N.a.	HP	2 HPs	*Vampirovibrio* spp.	≤ 2e-25
51	WMU93246.1	Sensor domain-containing diguanylate cyclase	HP	GGDEF domain-containing protein	*V. chlorellavorus*	0
52	WMU93247.1	N.a.	Cysteine desulfurase	Aminotransferase class V-fold PLP-dependent enzyme	*V. chlorellavorus*	0
53	WMU93248.1	N.a.	HP	Cysteine dioxygenase	*V. chlorellavorus*	2e-106
54	WMU93249.1	N.a.	Heme chaperone	Radical SAM family heme chaperone HemW	*V. chlorellavorus*	0
55	WMU93250.1	N.a.	HP	HP	*V. chlorellavorus*	1e-34
56	WMU93251.1	N.a.	HP	2 HPs	*Vampirovibrio* spp.	≤ 1e-26
57	WMU93252.1	Isocitrate dehydrogenase, partial	Isocitrate dehydrogenase [NADP]	Isocitrate dehydrogenase (NADP(+))	*V. chlorellavorus*	0
58	WMU93253.1	N.a.	Aconitate hydratase A	Aconitate hydratase	*V. chlorellavorus*	0

59	WMU93254.1	Ribonucleoside-diphosphate reductase	Vitamin B12-dependent ribonucleoside-diphosphate reductase	Adenosylcobalamin-dependent ribonucleoside-diphosphate reductase	*V. chlorellavorus*	0
60	WMU93255.1	N.a.	D-inositol-3-phosphate glycosyltransferase	Glycosyltransferase family 4 protein	*V. chlorellavorus*	0
61	WMU93256.1	RNA polymerase sigma factor	RNA polymerase sigma factor	Sigma-70 family RNA polymerase sigma factor	*V. chlorellavorus*	0
62	WMU93257.1	N.a.	Photosystem I assembly protein	Tetratricopeptide repeat protein	*V. chlorellavorus*	0
63	WMU93258.1	N.a.	Ribonuclease Y	HDOD domain-containing protein	*V. chlorellavorus*	0
64	WMU93259.1	N.a.	HP	2 HPs	*Vampirovibrio* spp.	≤ 1e-89
65	WMU93260.1	N.a.	HP	Flagellar filament capping protein FliD	*V. chlorellavorus*	0
66	WMU93261.1	N.a.	Putative amino acid permease	Aminoacid permease	*V. chlorellavorus*	0
67	WMU93262.1	N.a.	HP	2 HPs	*Vampirovibrio* spp.	≤ 7e-27
68	WMU93263.1	N.a.	tRNA-specific 2-thiouridylase	tRNA 2-thiouridine(34) synthase MnmA	*V. chlorellavorus*	0
69	WMU93264.1	N.a.	HP	Pentapeptide repeat-containing protein	*V. chlorellavorus*	0
70	WMU93265.1	N.a.	HP	M2 family metallopeptidase	*V. chlorellavorus*	0
71	WMU93266.1	N.a.	HP	2 HPs	*Vampirovibrio* spp.	≤ 2e-61
72	WMU93267.1	Bifunctional NAD-dependent-3-hydroxypropionate dehydrogenase	Putative oxidoreductase	SDR family oxidoreductase	*V. chlorellavorus*	0
73	WMU93268.1	N.a.	HP	2 HPs	*Vampirovibrio* spp.	≤ 4e-78
74	WMU93269.1	N.a.	HP	3 HPs	*Vampirovibrio* spp.	≤ 1e-04
75	WMU93270.1	N.a.	HP	HP	*V. chlorellavorus*	8e-15
76	WMU93271.1	N.a.	HP	DUF3228 family protein	*V. chlorellavorus*	8e-106
77	WMU93272.1	DNA polymerase IV	DNA polymerase IV	DNA polymerase IV	*V. chlorellavorus*	0
78	WMU93273.1	N.a.	HP	HP	*V. chlorellavorus*	4e-23
79	WMU93274.1	N.a.	HP	2 HPs	*Vampirovibrio* spp.	≤ 2e-20
80	WMU93275.1	N.a.	HP	2 HPs	*Vampirovibrio* spp.	≤ 7e-54
81	WMU93276.1	PrsA Phosphoribosyl-pyrophosphate synthetase	Ribose-phosphate pyrophosphokinase	Ribose-phosphate diphosphokinase	*V. chlorellavorus*	0
82	WMU93277.1	N.a.	HP	AtaL-like protein	*V. chlorellavorus*	1e-102
83	WMU93278.1	N.a.	HP	HP	*V. chlorellavorus*	1e-139
84	WMU93279.1	N.a.	Anthranilate synthase component 2	Anthranilate synthase component II	*V. chlorellavorus*	1e-119
85	WMU93280.1	N.a.	HP	HP	*V. chlorellavorus*	2e-73
86	WMU93281.1	N.a.	HP	2 HPs	*Vampirovibrio* spp.	≤ 3e-54
87	WMU93282.1	N.a.	HP	2 HPs	*Vampirovibrio* spp.	0
88	WMU93283.1	N.a.	HP	ABC transporter permease	*V. chlorellavorus*	0
89	WMU93284.1	N.a.	Glutathione transport system permease protein	ABC transporter permease	*V. chlorellavorus*	0
90	WMU93285.1	N.a.	Acetyltransferase	GNAT family N-acetyltransferase	*V. chlorellavorus*	1e-94
91	WMU93286.1	N.a.	Putative protein YccU	CoA-binding protein	*V. chlorellavorus*	4e-85
92	WMU93287.1	N.a.	HP	HP	*V. chlorellavorus*	9e-157

93	WMU93288.1	3-polyprenyl-4-hydroxybenzoate carboxy-lyase	Putative UbiX-like flavin prenyltransferase	UbiX family flavin prenyltransferase	*V. chlorellavorus*	6e-158
94	WMU93289.1	N.a.	HP	2 HPs	*Vampirovibrio* spp.	≤ 1e-61
95	WMU93290.1	PcnB tRNA nucleotidyl-transferase/poly(A) polymerase	Poly(A) polymerase I	CCA tRNA nucleotidyltransferase	*Desulfobacula* sp.	3e-42
96	WMU93291.1	N.a.	ATP-dependent dethiobiotin synthetase	AAA family ATPase	*V. chlorellavorus*	1e-168
97	WMU93292.1	N.a.	Lipid II flippase	Murein biosynthesis integral membrane protein MurJ	*V. chlorellavorus*	0
98	WMU93293.1	Proline dehydrogenase	Proline dehydrogenase 1	Proline dehydrogenase family protein	*V. chlorellavorus*	0
99	WMU93294.1	N.a.	Acyl-CoA dehydrogenase	Acyl-CoA dehydrogenase family protein	*V. chlorellavorus*	0
100	WMU93295.1	N.a.	Chemotaxis protein	Motility protein A	*V. chlorellavorus*	4e-180
101	WMU93296.1	N.a.	Outer membrane protein A	OmpA/MotB family protein	*V. chlorellavorus*	3e-152
102	WMU93297.1	N.a.	HP	Flagellar basal body-associated FliL family protein	*V. chlorellavorus*	4e-153
103	WMU93298.1	N.a.	Flagellar motor switch protein	Flagellar motor switch protein FliM	*V. chlorellavorus*	0
104	WMU93299.1	N.a.	HP	Flagellar motor switch phosphatase FliY	*V. chlorellavorus*	0
105	WMU93300.1	N.a.	Secreted effector protein	Pentapeptide repeat-containing protein	*V. chlorellavorus*	7e-169
106	WMU93301.1	N.a.	HP	Metallothionein	*V. chlorellavorus*	8e-45
107	WMU93302.1	Enoyl-[acyl-carrier-protein] reductase [NADH]	Enoyl-[acyl-carrier-protein] reductase [NADH]	Enoyl-ACP reductase FabI	*V. chlorellavorus*	0
108	WMU93303.1	GTP-binding protein	Putative GTP-binding protein	Ribosome biogenesis GTP-binding protein YihA/YsxC	*V. chlorellavorus*	9e-151
109	WMU93304.1	N.a.	HP	SH3 domain-containing protein	*Geomonas oryzisoli*	8e-05
110	WMU93305.1	N.a.	HP	2 HPs	*Vampirovibrio* spp.	≤ 4e-21
111	WMU93306.1	N.a.	HP	2 HPs	*Vampirovibrio* spp.	≤ 2e-52
112	WMU93307.1	N.a.	HP	DUF366 family protein	*V. chlorellavorus*	8e-138
113	WMU93308.1	Penicillin-insensitive trans-glycosylase & transpeptidase	Biosynthetic peptidoglycan transglycosylase	Transglycosylase domain-containing protein	*V. chlorellavorus*	0
114	WMU93309.1	Glycine-tRNA ligase beta subunit	Glycine--tRNA ligase beta subunit	Glycine--tRNA ligase subunit beta	*V. chlorellavorus*	0
115	WMU93310.1	N.a.	Lipid II isoglutaminyl synthase (glutamine-hydrolyzing) subunit	Type 1 glutamine amidotransferase	*V. chlorellavorus*	7e-160
116	WMU93311.1	N.a.	HP	2 HPs	*Vampirovibrio* spp.	≤ 9e-22
117	WMU93312.1	N.a.	HP	2 HPs	*Vampirovibrio* spp.	≤ 3e-43
118	WMU93313.1	N.a.	HP	TIGR04552 family protein	*V. chlorellavorus*	0
119	WMU93314.1	N.a.	Protein-export membrane protein	Protein translocase subunit SecF	*V. chlorellavorus*	0
120	WMU93315.1	N.a.	Protein translocase subunit	Protein translocase subunit SecD	*V. chlorellavorus*	0
121	WMU93316.1	Tail fibers protein	HP	Phosphodiester glycosidase family protein	*V. chlorellavorus*	0
122	WMU93317.1	N.a.	HP	2 HPs	*Vampirovibrio* spp.	≤ 1e-95
123	WMU93318.1	N.a.	HP	2 HPs	*Vampirovibrio spp.*	≤ 7e-102
124	WMU93319.1	N.a.	Holo-[acyl-carrier-protein] synthase	Holo-ACP synthase	*V. chlorellavorus*	5e-90

125	WMU93320.1	N.a.	HP	2 HPs	*Vampirovibrio* spp.	≤ 3e-56
126	WMU93321.1	N.a.	HP	Serine/threonine-protein kinase	Unclassified *Streptosporangium*	1e-12
127	WMU93322.1	N.a.	HP	2 HPs	*Vampirovibrio* spp.	≤ 1e-151
128	WMU93323.1	N.a.	HP	2 HPs	*Vampirovibrio* spp.	≤ 1e-35
129	WMU93324.1	N.a.	HP	AbrB/MazE/SpoVT family DNA-binding domain-containing protein	*V. chlorellavorus*	1e-09
130	WMU93325.1	N.a.	HP	Type II toxin-antitoxin system PemK/MazF family toxin	*Ekhidna* sp.	1e-15
131	WMU93326.1	N.a.	Segregation and condensation protein B	SMC-Scp complex subunit ScpB	*V. chlorellavorus*	6e-155
132	WMU93327.1	N.a.	Segregation and condensation protein A	Segregation and condensation protein A	*V. chlorellavorus*	0
133	WMU93328.1	DNA repair exonuclease SbcCD ATPase subunit	Chromosome partition protein	Chromosome segregation protein SMC	*V. chlorellavorus*	0
134	WMU93329.1	N.a.	Putative zinc protease	M16 family metallopeptidase	*V. chlorellavorus*	0
135	WMU93330.1	Putative Zn-dependent peptidase	Putative zinc protease	M16 family metallopeptidase	*V. chlorellavorus*	0
136	WMU93331.1	N.a.	HP	S8 family serine peptidase	*V. chlorellavorus*	0
137	WMU93332.1	N.a.	HP	None	None	N.a.
138	WMU93333.1	GIY-YIG nuclease superfamily protein	HP	GIY-YIG nuclease family protein	*V. chlorellavorus*	6e-54
139	WMU93334.1	N.a.	Cardiolipin synthase A	Phospholipase D-like domain-containing protein	*V. chlorellavorus*	0
140	WMU93335.1	N.a.	HP	2 HPs	*Vampirovibrio* spp.	≤ 1e-22
141	WMU93336.1	N.a.	HP	Type II secretion system protein	*Vampirovibrio* sp.	1e-52
142	WMU93337.1	N.a.	HP	Type II secretion system protein	*Vampirovibrio* sp.	1e-45
143	WMU93338.1	N.a.	UvrABC system protein A	Excinuclease ABC subunit UvrA	*V. chlorellavorus*	0
144	WMU93339.1	N.a.	HP	2 HPs	*Vampirovibrio* spp.	≤ 1e-109
145	WMU93340.1	N.a.	HP	2 HPs	*Vampirovibrio* spp.	≤ 3e-88
146	WMU93341.1	MutT-like nucleotide pyrophosphohydrolase	RNA pyrophosphohydrolase	NUDIX domain-containing protein	*V. chlorellavorus*	2e-100
147	WMU93342.1	N.a.	HP	2 HPs	*Vampirovibrio* spp.	≤ 1e-06
148	WMU93343.1	N.a.	HP	2 HPs	*Vampirovibrio* spp.	≤ 5e-69
149	WMU93344.1	N.a.	UDP-N-acetylglucosamine 2-epimerase	Non-hydrolyzing UDP-N-acetylglucosamine 2-epimerase	*V. chlorellavorus*	0
150	WMU93345.1	N.a.	HP	TonB family protein	*V. chlorellavorus*	1e-180
151	WMU93346.1	TopA topoisomerase IA	DNA topoisomerase 1	Type I DNA topoisomerase	*V. chlorellavorus*	0
152	WMU93347.1	N.a.	HP	S-layer homology domain-containing protein	*Vampirovibrio* sp.	2e-19
153	WMU93348.1	N.a.	Photosystem II 12 kDa extrinsic protein	ComEA family DNA-binding protein	*V. chlorellavorus*	6e-58
154	WMU93349.1	N.a.	HP	2 HPs	*Vampirovibrio* spp.	≤ 4e-82
155	WMU93350.1	N.a.	HP	YggT family protein	*V. chlorellavorus*	4e-58

156	WMU93351.1	N.a.	S-methyl-5'-thioadenosine phosphorylase	S-methyl-5'-thioadenosine phosphorylase	*V. chlorellavorus*	0
157	WMU93352.1	N.a.	HP	HP	*V. chlorellavorus*	0
158	WMU93353.1	N.a.	HP	2 HPs	*Vampirovibrio* spp.	≤ 6e-15
159	WMU93354.1	N.a.	Cobalt/magnesium transport protein	Magnesium/cobalt transporter CorA	*V. chlorellavorus*	0
160	WMU93355.1	N.a.	HP	2 HPs	*Vampirovibrio* spp.	≤ 4e-53
161	WMU93356.1	N.a.	HP	DUF2145 domain-containing protein	*V. chlorellavorus*	1e-164
162	WMU93357.1	N.a.	HP	4 HPs	*Vampirovibrio* spp.	≤ 3e-14
163	WMU93358.1	N.a.	HP	2 HPs	*Vampirovibrio* spp.	≤ 3e-37
164	WMU93359.1	N.a.	Mannosylglucosyl-3-phosphoglycerate phosphatase	Bifunctional metallophosphatase/5'-nucleotidase	*V. chlorellavorus*	0
165	WMU93360.1	N.a.	Zinc uptake regulation protein	Fur family transcriptional regulator	*V. chlorellavorus*	1e-94
166	WMU93361.1	N.a.	HP	RNA recognition motif domain-containing protein	*V. chlorellavorus*	2e-114
167	WMU93362.1	N.a.	HP	4 HPs	*Vampirovibrio* spp.	≤ 4e-08
168	WMU93363.1	N.a.	HP	Glycosyl hydrolase family 8	*V. chlorellavorus*	0
169	WMU93364.1	N.a.	HP	Sugar transferase	*V. chlorellavorus*	2e-142
170	WMU93365.1	N.a.	HP	HP	*V. chlorellavorus*	6e-136
171	WMU93366.1	N.a.	HP	204 HPs	Multiple species	≤ 4e-06
172	WMU93367.1	N.a.	Methylthioribulose-1-phosphate dehydratase	Methylthioribulose 1-phosphate dehydratase	*V. chlorellavorus*	1e-138
173	WMU93368.1	N.a.	Acireductone dioxygenase	1,2-dihydroxy-3-keto-5-methylthiopentene dioxygenase	*V. chlorellavorus*	4e-121
174	WMU93369.1	N.a.	Enolase-phosphatase E1	Acireductone synthase	*V. chlorellavorus*	4e-149
175	WMU93370.1	Aldehyde dehydrogenase B	Succinate-semialdehyde dehydrogenase [NADP(+)]	Aldehyde dehydrogenase family protein	*V. chlorellavorus*	0
176	WMU93371.1	N.a.	HP	ABC transporter substrate-binding protein	*V. chlorellavorus*	0
177	WMU93372.1	N.a.	HP	2 HPs	*Vampirovibrio* spp.	≤ 3e-44
178	WMU93373.1	N.a.	Putative metal-dependent hydrolase	TatD family hydrolase	*V. chlorellavorus*	0
179	WMU93374.1	N.a.	HP	DUF6980 family protein	*V. chlorellavorus*	3e-120
180	WMU93375.1	Leucine-tRNA ligase	Methionine--tRNA ligase	Methionine--tRNA ligase	*V. chlorellavorus*	0
181	WMU93376.1	N.a.	HP	None	None	N.a.
182	WMU93377.1	N.a.	Threonylcarbamoyladenosine tRNA methylthiotransferase	tRNA (N(6)-L-threonylcarbamoyladenosine(37)-C(2))-methylthiotransferase MtaB	*V. chlorellavorus*	0
183	WMU93378.1	N.a.	Nickel-responsive regulator	CopG family ribbon-helix-helix protein	*V. chlorellavorus*	2e-29
184	WMU93379.1	N.a.	Nickel-responsive regulator	CopG family ribbon-helix-helix protein	*V. chlorellavorus*	2e-32
185	WMU93380.1	N.a.	HP	2 HPs	*Vampirovibrio* spp.	≤ 4e-151
186	WMU93381.1	PurB Adenylosuccinate lyase	Adenylosuccinate lyase	Adenylosuccinate lyase	*V. chlorellavorus*	0
187	WMU93382.1	N.a.	HP	Mechanosensitive ion channel family protein	*V. chlorellavorus*	0

188	WMU93383.1	N.a.	Glycogen operon protein	Glycogen debranching protein GlgX	*V. chlorellavorus*	0
189	WMU93384.1	N.a.	HP	MerR family transcriptional regulator	*V. chlorellavorus*	1e-124
190	WMU93385.1	Cell division protease	ATP-dependent zinc metalloprotease	ATP-dependent zinc metalloprotease FtsH	*V. chlorellavorus*	0
191	WMU93386.1	Threonine-tRNA ligase	Proline--tRNA ligase	Proline--tRNA ligase	*V. chlorellavorus*	0
192	WMU93387.1	FusA Translation elongation factors (GTPases)	Elongation factor 4	Translation elongation factor 4	*V. chlorellavorus*	0
193	WMU93388.1	N.a.	HP	HP	*Vampirovibrio* sp.	2e-28
194	WMU93389.1	N.a.	HP	2 HPs	*Vampirovibrio* spp.	≤ 2e-38
195	WMU93390.1	Peptidase M15	HP	D-Ala-D-Ala carboxypeptidase family metallohydrolase	*V. chlorellavorus*	9e-82
196	WMU93391.1	N.a.	HP	2 HPs	*Vampirovibrio* spp.	≤ 1e-41
197	WMU93392.1	N.a.	HP	2 HPs	*Vampirovibrio* spp.	≤ 8e-53
198	WMU93393.1	N.a.	HP	Phage tail protein	*Pseudomonas* sp. NFACC36	3e-10
199	WMU93394.1	N.a.	HP	17 HPs	Multiple species	<6e-04
200	WMU93395.1	N.a.	HP	Phage adaptor protein	*V. chlorellavorus*	5e-134
201	WMU93396.1	N.a.	HP	2 HPs	*Vampirovibrio* spp.	≤ 1e-31
202	WMU93397.1	Capsid protein	HP	P22 phage major capsid protein family protein	*V. chlorellavorus*	0
203	WMU93398.1	N.a.	HP	2 HPs	*Vampirovibrio* spp.	≤ 6e-74
204	WMU93399.1	N.a.	HP	2 HPs	*Vampirovibrio* spp.	≤ 1e-45
205	WMU93400.1	Portal protein	HP	Portal protein	*V. chlorellavorus*	0
206	WMU93401.1	N.a.	HP	3 HPs	*Vampirovibrio* spp.	≤ 4e-19
207	WMU93402.1	N.a.	Potassium transporter	APC family permease	*V. chlorellavorus*	0
208	WMU93403.1	Phosphoglucomutase	Phosphoglucomutase	Phosphoglucomutase/phosphomannomutase family protein	*Leptolyngbya* sp. FACHB-261	3e-103
209	WMU93404.1	N.a.	K(+)-insensitive pyrophosphate-energized proton pump	Sodium-translocating pyrophosphatase	*V. chlorellavorus*	0
210	WMU93405.1	N.a.	Nickel-responsive regulator	CopG family ribbon-helix-helix protein	*V. chlorellavorus*	2e-25
211	WMU93406.1	N.a.	HP	Flagellar brake protein	*V. chlorellavorus*	2e-112
212	WMU93407.1	RNA polymerase sigma-W factor	ECF RNA polymerase sigma factor	Sigma-70 family RNA polymerase sigma factor	*V. chlorellavorus*	7e-145
213	WMU93408.1	N.a.	HP	2 HPs	*Vampirovibrio* spp.	≤ 4e-96
214	WMU93409.1	N.a.	HP	Spy/CpxP family protein refolding chaperone	*V. chlorellavorus*	3e-89
215	WMU93410.1	LysU Lysyl-tRNA synthetase (class II)	Aspartate--tRNA ligase	Aspartate--tRNA ligase	*V. chlorellavorus*	0
216	WMU93411.1	BaeS Signal transduction histidine kinase	Adaptive-response sensory-kinase	GAF domain-containing protein	*V. chlorellavorus*	0
217	WMU93412.1	N.a.	Dephospho-CoA kinase	Dephospho-CoA kinase	*V. chlorellavorus*	8e-141
218	WMU93413.1	N.a.	HP	S-layer homology domain-containing protein	*Vallitalea pronyensis*	7e-04

219	WMU93414.1	N.a.	Ribonuclease HIII	Ribonuclease HIII	*V. chlorellavorus*	0
220	WMU93415.1	N.a.	HP	DUF2730 domain-containing protein	*Myxococcus* sp. XM-1-1-1	2e-15
221	WMU93416.1	N.a.	HP	2 HPs	*Vampirovibrio* spp.	≤ 4e-39
222	WMU93417.1	N.a.	Glutamyl-tRNA(Gln) amidotransferase subunit A	Asp-tRNA(Asn)/Glu-tRNA(Gln) amidotransferase subunit GatA	*V. chlorellavorus*	0
223	WMU93418.1	N.a.	Bifunctional homocysteine S-methyltransferase/5	Methylenetetrahydrofolate reductase	*V. chlorellavorus*	0
224	WMU93419.1	N.a.	HP	2 HPs	*Vampirovibrio* spp.	≤ 4e-75
225	WMU93420.1	N.a.	HP	DUF5522 domain-containing protein	*V. chlorellavorus*	2e-37
226	WMU93421.1	Amidophosphoribosyltransferase	Amidophosphoribosyltransferase	Amidophosphoribosyltransferase	*V. chlorellavorus*	0
227	WMU93422.1	Putative phosphoribosyl formylglycinamidine (FGAM) synthase II	Phosphoribosylformylglycinamidine synthase subunit	Phosphoribosylformylglycinamidine synthase subunit PurL	*V. chlorellavorus*	0
228	WMU93423.1	N.a.	HP	Bax inhibitor-1 family protein	*V. chlorellavorus*	1e-121
229	WMU93424.1	Pentapeptide repeat family protein	HP	Pentapeptide repeat-containing protein	*V. chlorellavorus*	6e-139
230	WMU93425.1	N.a.	HP	HP	*V. chlorellavorus*	2e-26
231	WMU93426.1	SpeB Arginase/agmatinase/ formimionoglutamate hydrolase, arginase family	Agmatinase	Agmatinase	*V. chlorellavorus*	0
232	WMU93427.1	SpeE Spermidine synthase	Polyamine aminopropyltransferase	Polyamine aminopropyltransferase	*V. chlorellavorus*	0
233	WMU93428.1	SpeD S-adenosylmethionine decarboxylase	S-adenosylmethionine decarboxylase proenzyme	S-adenosylmethionine decarboxylase	*V. chlorellavorus*	3e-97
234	WMU93429.1	N.a.	S-adenosylmethionine decarboxylase proenzyme	Adenosylmethionine decarboxylase	*V. chlorellavorus*	3e-113
235	WMU93430.1	Bactoprenol glucosyl transferase	Dodecaprenyl-phosphate galacturonate synthase	Glycosyltransferase family 2 protein	*V. chlorellavorus*	4e-172
236	WMU93431.1	N.a.	HP	Phospholipid carrier-dependent glycosyltransferase	*Kordia* sp.	3e-17
237	WMU93432.1	N.a.	Guanine deaminase	Guanine deaminase	*V. chlorellavorus*	0
238	WMU93433.1	N.a.	HP	2 HPs	*V. chlorellavorus*	≤ 2e-52
239	WMU93434.1	ATP-dependent protease gp262	HP	MgtC/SapB family protein	*V. chlorellavorus*	1e-173
240	WMU93435.1	N.a.	HP	Aminotransferase class IV	*V. chlorellavorus*	2e-165
241	WMU93436.1	N.a.	HP	ATP-dependent endonuclease	*V. chlorellavorus*	0
242	WMU93437.1	N.a.	HP	Ribosomal protein S18-alanine N-acetyltransferase	*V. chlorellavorus*	2e-136
243	WMU93438.1	N.a.	HP	Biotin transporter BioY	*V. chlorellavorus*	9e-158
244	WMU93439.1	N.a.	5-oxoprolinase subunit A	LamB/YcsF family protein	*V. chlorellavorus*	0
245	WMU93440.1	RNA binding protein	Nucleotide-binding protein	RNase adapter RapZ	*V. chlorellavorus*	0
246	WMU93441.1	N.a.	Gluconeogenesis factor	Gluconeogenesis factor YvcK family protein	*V. chlorellavorus*	0
247	WMU93442.1	N.a.	3-methyl-2-oxobutanoate hydroxymethyltransferase	3-methyl-2-oxobutanoate hydroxymethyltransferase	*V. chlorellavorus*	0

248	WMU93443.1	N.a.	HP	HP	*V. chlorellavorus*	2e-127
249	WMU93444.1	N.a.	Pantothenate synthetase	Pantoate--beta-alanine ligase	*V. chlorellavorus*	0
250	WMU93445.1	N.a.	HP	2 HPs	*V. chlorellavorus*	≤ 3e-28
251	WMU93446.1	MerR family transcriptional regulator	HP	MerR family transcriptional regulator	*V. chlorellavorus*	6e-114
252	WMU93447.1	Iron-sulfur cluster assembly protein	FeS cluster assembly protein	Fe-S cluster assembly protein SufB	*V. chlorellavorus*	0
253	WMU93448.1	N.a.	Putative ATP-dependent transporter	Fe-S cluster assembly ATPase SufC	*V. chlorellavorus*	0
254	WMU93449.1	N.a.	FeS cluster assembly protein	Fe-S cluster assembly protein SufD	*V. chlorellavorus*	0
255	WMU93450.1	N.a.	Cysteine desulfurase	Cysteine desulfurase	*V. chlorellavorus*	0
256	WMU93451.1	N.a.	Iron-sulfur cluster assembly scaffold protein	Fe-S cluster assembly sulfur transfer protein SufU	*V. chlorellavorus*	1e-102

[a]Twelve ORFs annotated as 'unnamed protein product', 'pentapeptide', 'protein of unknown function', 'hypothetical protein' and 'gp' are listed as N.a.

[b]The top functionally annotated hit is listed. When no functionally annotated homologs were detected, the number of significant ($E < 0.001$) HP hits is shown instead.

Abbreviations: ORF, open reading frame; N.a., not annotated; HP, hypothetical protein.

Table S2. InterPro families and Foldseek top hits of PhiMa05 ribosomal proteins.

ORF	GenBank accession	InterPro family name	InterPro family ID	pTM/ eTM[a]	Foldseek top hit	PDB ID	Organism	Foldseek *E*-value	Foldseek Prob.
12	WMU93207.1	Small ribosomal subunit protein bS1-like	IPR050437	0.85	30S ribosomal protein S1	7A05-A	*Vibrio vulnificus*	3.25e-16	1
13	WMU93208.1	Large ribosomal subunit protein bL21	IPR001787	0.84	50S ribosomal protein L21	7NHN-U	*Listeria monocytogenes* EGD-e	4.11e-11	1
14	WMU93209.1	Large ribosomal subunit protein bL27	IPR001684	0.81	50S ribosomal protein L27	8BUU-W	*Bacillus subtilis* subsp. *subtilis* str. 168	1.60e-10	1
35	WMU93230.1	Large ribosomal subunit protein bL33	IPR001705	0.59	50S ribosomal protein L33	8CVM-0	*Cutibacterium acnes*	2.11e-4	1
38	WMU93233.1	Ribosomal protein uL11	IPR000911	0.87	50S ribosomal protein L11	5O61-J	*Mycolicibacterium smegmatis* MC2 155	1.15e-16	1
39	WMU93234.1	Large ribosomal subunit protein uL1, bacterial-type	IPR005878	0.91	50S ribosomal protein L1	7P7T-F	*Enterococcus faecalis*	2.21e-31	1
108	WMU93303.1	GTP-binding protein, ribosome biogenesis, YsxC	IPR019987	0.86	GTP-binding protein YsxC	1SUL-B	*B. subtilis*	1.89e-22	1
242	WMU93437.1	N-acetyltransferase RimI/Ard1	IPR006464	0.89	Ribosomal-protein-alanine acetyltransferase	5ISV-B	*Escherichia coli* O157:H7	1.02e-13	1

[a]AlphaFold pTM is shown for all ORFs except ORF 12 for which D-I-TASSER eTM is shown.

Abbreviations: ORF, open reading frame; pTM, predicted template modelling score; eTM, estimated template modelling score; Prob., Foldseek probability of true-positive match.

Table S3. InterPro families and Foldseek top hits of PhiMa05 hypothetical proteins.

ORF	GenBank accession	InterPro family name	InterPro family ID	pTM	Foldseek top hit	PDB ID	Organism	Foldseek *E*-value	Foldseek Prob.
2	WMU93197.1	None	None	0.37	N.a.	N.a.	N.a.	N.a.	N.a.
5	WMU93200.1	None	None	0.61	Adenylyl cyclase	5D15-B	*Mycobacterium avium* subsp. *avium* 10-9275	1.21e-08	1
6	WMU93201.1	None	None	0.27	N.a.	N.a.	N.a.	N.a.	N.a.
8	WMU93203.1	None	None	0.5	NS	NS	NS	NS	NS
15	WMU93210.1	None	None	0.18	N.a.	N.a.	N.a.	N.a.	N.a.
19	WMU93214.1	None	None	0.29	N.a.	N.a.	N.a.	N.a.	N.a.
28	WMU93223.1	None	None	0.42	N.a.	N.a.	N.a.	N.a.	N.a.
30	WMU93225.1	None	None	0.21	N.a.	N.a.	N.a.	N.a.	N.a.
33	WMU93228.1	None	None	0.55	NS	NS	NS	NS	NS
50	WMU93245.1	None	None	0.37	N.a.	N.a.	N.a.	N.a.	N.a.
55	WMU93250.1	None	None	0.32	N.a.	N.a.	N.a.	N.a.	N.a.
56	WMU93251.1	None	None	0.37	N.a.	N.a.	N.a.	N.a.	N.a.
62	WMU93257.1	Ycf3/AcsC/BcsC/TPR Multifunctional	IPR051685	0.52	NS	NS	NS	NS	NS
64	WMU93259.1	None	None	0.51	KDP operon transcriptional regulatory protein KdpE	4KFC-B	*Escherichia coli* K-12	3.68e-05	1
67	WMU93262.1	None	None	0.68	NS	NS	NS	NS	NS
69	WMU93264.1	Thylakoid lumenal 17.4 kDa protein, chloroplastic	PTHR47485	0.93	Beta1 solenoid	4YC5-A	Synthetic	1.18e-13	1
71	WMU93266.1	None	None	0.67	NS	NS	NS	NS	NS
73	WMU93268.1	None	None	0.43	N.a.	N.a.	N.a.	N.a.	N.a.
74	WMU93269.1	None	None	0.66	NS	NS	NS	NS	NS
75	WMU93270.1	None	None	0.16	N.a.	N.a.	N.a.	N.a.	N.a.
76	WMU93271.1	Protein of unknown function DUF3228	IPR021610	0.9	HP	2PD0-D	*Cryptosporidium parvum*	7.07e-12	1
78	WMU93273.1	None	None	0.14	N.a.	N.a.	N.a.	N.a.	N.a.
79	WMU93274.1	None	None	0.56	NS	NS	NS	NS	NS
80	WMU93275.1	None	None	0.33	N.a.	N.a.	N.a.	N.a.	N.a.
83	WMU93278.1	None	None	0.23	N.a.	N.a.	N.a.	N.a.	N.a.
85	WMU93280.1	None	None	0.29	N.a.	N.a.	N.a.	N.a.	N.a.
86	WMU93281.1	None	None	0.22	N.a.	N.a.	N.a.	N.a.	N.a.
87	WMU93282.1	None	None	0.52	None	None	None	None	None
92	WMU93287.1	None	None	0.63	NS	NS	NS	NS	NS
94	WMU93289.1	None	None	0.61	NS	NS	NS	NS	NS

105	WMU93300.1	Pentapeptide repeat and BTB/POZ domain-containing protein	IPR051082	0.84	Beta1 solenoid	4YCQ-A-2	Synthetic	1.95e-12	1
110	WMU93305.1	None	None	0.4	N.a.	N.a.	N.a.	N.a.	N.a.
111	WMU93306.1	None	None	0.2	N.a.	N.a.	N.a.	N.a.	N.a.
112	WMU93307.1	Protein of unknown function DUF366; Class II Aminoacyl-tRNA synthetase/Biotinyl protein ligase (BPL) and lipoyl protein ligase (LPL)	IPR007162; IPR045864	0.92	HP	2DDZ-F	*Pyrococcus horikoshii* OT3	1.18e-19	1
116	WMU93311.1	None	None	0.53	NS	NS	NS	NS	NS
117	WMU93312.1	None	None	0.32	N.a.	N.a.	N.a.	N.a.	N.a.
118	WMU93313.1	Uncharacterised protein family CHP04562; Nucleotidyltransferase superfamily	IPR030824; IPR043519	0.78	GTP pyrophosphokinase YjbM	5DEC-B	*Bacillus subtilis* subsp. *subtilis* str. 168	1.13e-04	1
122	WMU93317.1	None	None	0.46	N.a.	N.a.	N.a.	N.a.	N.a.
123	WMU93318.1	None	None	0.48	N.a.	N.a.	N.a.	N.a.	N.a.
125	WMU93320.1	Histone H1-like nucleoprotein HC2	IPR009970	0.2	N.a.	N.a.	N.a.	N.a.	N.a.
127	WMU93322.1	None	None	0.31	N.a.	N.a.	N.a.	N.a.	N.a.
128	WMU93323.1	None	None	0.34	N.a.	N.a.	N.a.	N.a.	N.a.
137	WMU93332.1	None	None	0.22	N.a.	N.a.	N.a.	N.a.	N.a.
140	WMU93335.1	None	None	0.38	N.a.	N.a.	N.a.	N.a.	N.a.
144	WMU93339.1	None	None	0.69	NS	NS	NS	NS	NS
145	WMU93340.1	None	None	0.35	N.a.	N.a.	N.a.	N.a.	N.a.
147	WMU93342.1	None	None	0.14	N.a.	N.a.	N.a.	N.a.	N.a.
148	WMU93343.1	None	None	0.2	N.a.	N.a.	N.a.	N.a.	N.a.
154	WMU93349.1	None	None	0.56	NS	NS	NS	NS	NS
157	WMU93352.1	None	None	0.58	NS	NS	NS	NS	NS
158	WMU93353.1	Prokaryotic membrane lipoprotein lipid attachment site profile	PS51257	0.5	NS	NS	NS	NS	NS
160	WMU93355.1	None	None	0.69	NS	NS	NS	NS	NS
161	WMU93356.1	Uncharacterised conserved protein UCP028477	IPR014547	0.71	NS	NS	NS	NS	NS
162	WMU93357.1	None	None	0.43	N.a.	N.a.	N.a.	N.a.	N.a.
163	WMU93358.1	None	None	0.68	NS	NS	NS	NS	NS
167	WMU93362.1	None	None	0.56	NS	NS	NS	NS	NS
170	WMU93365.1	None	None	0.17	N.a.	N.a.	N.a.	N.a.	N.a.
171	WMU93366.1	None	None	0.83	NS	NS	NS	NS	NS
177	WMU93372.1	None	None	0.83	NS	NS	NS	NS	NS

179	WMU93374.1	Domain of unknown function DUF6980	IPR053918	0.81	NS	NS	NS	NS	NS
181	WMU93376.1	None	None	0.23	N.a.	N.a.	N.a.	N.a.	N.a.
185	WMU93380.1	None	None	0.55	Flagella basal-body protein	3W1E-A	*Vibrio alginolyticus*	5.42e-06	1
193	WMU93388.1	None	None	0.22	N.a.	N.a.	N.a.	N.a.	N.a.
194	WMU93389.1	None	None	0.48	N.a.	N.a.	N.a.	N.a.	N.a.
196	WMU93391.1	None	None	0.37	N.a.	N.a.	N.a.	N.a.	N.a.
197	WMU93392.1	None	None	0.57	NS	NS	NS	NS	NS
199	WMU93394.1	None	None	0.91	Packaged DNA stabilization protein gp10	8EAP-G	Salmonella phage P22	7.59e-19	1
201	WMU93396.1	None	None	0.6	NS	NS	NS	NS	NS
203	WMU93398.1	None	None	0.37	N.a.	N.a.	N.a.	N.a.	N.a.
204	WMU93399.1	None	None	0.38	N.a.	N.a.	N.a.	N.a.	N.a.
206	WMU93401.1	None	None	0.76	NS	NS	NS	NS	NS
213	WMU93408.1	None	None	0.21	N.a.	N.a.	N.a.	N.a.	N.a.
220	WMU93415.1	None	None	0.72	NS	NS	NS	NS	NS
221	WMU93416.1	None	None	0.44	N.a.	N.a.	N.a.	N.a.	N.a.
224	WMU93419.1	None	None	0.62	Putative lipoprotein B	2JXP-A	*Nitrosomonas europaea* ATCC 19718	4.61e-04	1
225	WMU93420.1	Universal ribosomal protein uL14; Protein of unknown function DUF5522	PTHR21037; IPR040807	0.67	NS	NS	NS	NS	NS
229	WMU93424.1	Thylakoid lumenal 17.4 kDa protein, chloroplastic	PTHR47485	0.89	Beta1 solenoid	4YCQ-A-2	Synthetic	1.26e-11	1
230	WMU93425.1	None	None	0.25	N.a.	N.a.	N.a.	N.a.	N.a.
238	WMU93433.1	None	None	0.23	N.a.	N.a.	N.a.	N.a.	N.a.
248	WMU93443.1	None	None	0.34	N.a.	N.a.	N.a.	N.a.	N.a.
250	WMU93445.1	None	None	0.35	N.a.	N.a.	N.a.	N.a.	N.a.

Abbreviations: ORF, open reading frame; pTM, predicted template modelling score; Prob., Foldseek probability of true-positive match; N.a., not applicable; NS, not significant.
